# Two salts of the 6,6-di­fluoro-6*H*-dibenzo[*c*,*e*][1,2]oxaborinin-6-ide anion with different cations

**DOI:** 10.1107/S2056989020015066

**Published:** 2020-11-17

**Authors:** Alexandra Budanow, Hans-Wolfram Lerner, Michael Bolte

**Affiliations:** a Institut für Anorganische und Analytische Chemie, Goethe-Universität Frankfurt, Max-von-Laue-Strasse 7, 60438 Frankfurt am Main, Germany

**Keywords:** crystal structure, boron, fluorine, potassium

## Abstract

The crystal structures are reported of the 6,6-di­fluoro-6*H*-dibenzo[*c*,*e*][1,2]oxaborinin-6-ide (or 9,9-di­fluoro-10-oxa-9-boraphenanthren-9-ide) anion with two different cations, namely, potassium 6,6-di­fluoro-6*H*-dibenzo[*c*,*e*][1,2]oxaborinin-6-ide, K^+^·C_12_H_8_BF_2_O^−^, (**II**), featuring a polymeric structure, and bis­(tetra­phenyl­phospho­nium) bis­(6,6-di­fluoro-6*H*-dibenzo[*c*,*e*][1,2]oxaborinin-6-ide) aceto­nitrile tris­olvate, 2C_24_H_20_P^+^·2C_12_H_8_BF_2_O^−^·3CH_3_CN, (**III**), which is composed of discrete cations, anions and aceto­nitrile solvent mol­ecules linked by C—H⋯O, C—H⋯N and C—H⋯F hydrogen bonds. There are only minor differences in the geometrical parameters of the anions in these structures.

## Abstract 2

The crystal structures are reported of the 6,6-di­fluoro-6*H*-dibenzo[*c*,*e*][1,2]oxaborinin-6-ide (or 9,9-di­fluoro-10-oxa-9-boraphenanthren-9-id)anion with two different cations, namely, potassium 6,6-di­fluoro-6*H*-dibenzo[*c*,*e*][1,2]oxaborinin-6-ide featuring a polymeric structure, and bis­(tetra­phenyl­phospho­nium) bis­(6,6-di­fluoro-6*H*-dibenzo[*c*,*e*][1,2]oxaborinin-6-ide) aceto­nitrile tris­olvate, which is composed of discrete cations, anions and aceto­nitrile solvent mol­ecules linked by C—H⋯O, C—H⋯N and C—H⋯F hydrogen bonds.

## Chemical context   

Oxabora­phenanthrenes are inter­esting building blocks for organic optoelectronic materials. Recently, we have prepared various 9-substituted oxabora­phenanthrene derivatives and investigated their stability and luminescence behavior (Budanow *et al.*, 2016 ▸). The starting material for our approach was 9-chloro-10,9-oxabora­phenanthrene (Budanow *et al.*, 2014 ▸), which is, however, an air-sensitive compound. Therefore we were now inter­ested in air-stable precursors for oxabora­phenanthrene preparation.
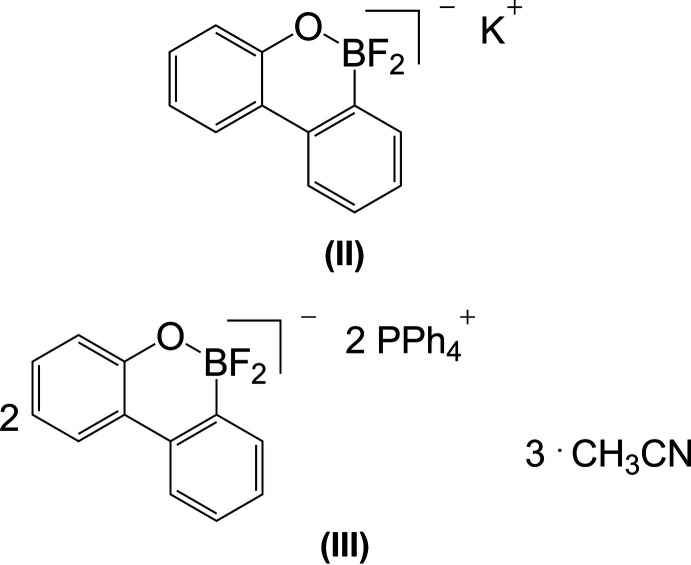


**Figure 1:**
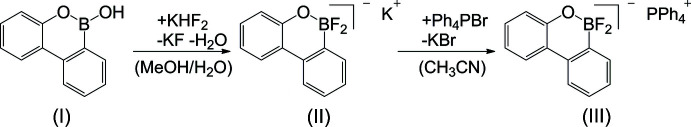
Synthesis of the 9,9-di­fluorido-10,9-oxabora­phenanthrene salts (**II**) and (**III**).

Because certain borinic acids are readily fluorinated with KHF_2_, we tested whether (**I**) (Fig. 1 ▸) could be transformed into a fluoro­borane upon treatment with KHF_2_. Indeed, by stirring a solution of (**I**) in the presence of KHF_2_ in methanol, a clean and qu­anti­tative OH/F exchange was established and 9,9-di­fluorido-10,9-oxabora­phenanthrene potassium (**II**) was obtained (Fig. 1 ▸). Subsequent treatment of (**II**) with Ph_4_PBr gave the 9,9-di­fluorido-10,9-oxabora­phenanthrene salt as an aceto­nitrile solvate (**III**). The crystal structures of (**II**) and (**III**) are now described.

## Structural commentary   

**Figure 2:**
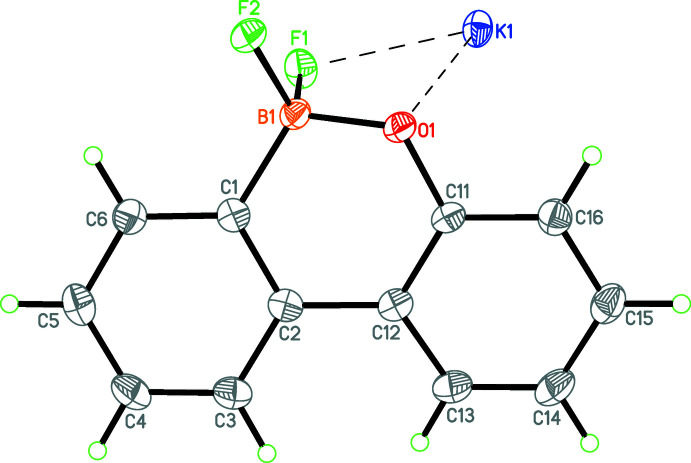
A perspective view of the asymmetric unit of (**II**). Displacement ellipsoids are drawn at the 50% probability level.

The anion in (**II**) has an almost planar skeleton (r.m.s. deviation 0.103 Å for the C, B and O atoms) (Fig. 2 ▸) and the bond lengths of the B and O atoms are in their usual ranges [B1—C1 = 1.591 (2); B1—O1 = 1.4564 (19), C11—O1 = 1.3678 (17) Å]. The B atom is substituted by two F ligands with bond lengths B1—F1 = 1.4483 (18) and B1—F2 = 1.4325 (18) Å. The dihedral angle between the C1–C6 and C11–C16 aromatic rings in the anion of (**II**) is 11.09 (9) Å.

**Figure 3:**
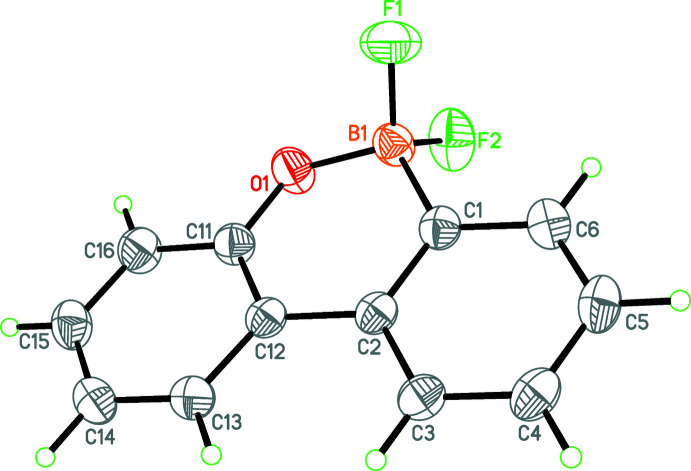
A perspective view of the first of the two anions in the asymmetric unit of (**III**). Displacement ellipsoids are drawn at the 50% probability level.

The asymmetric unit of (**III**) consists of two C_12_H_8_BF_2_O^−^ anions (Figs. 3 ▸ and 4 ▸), two C_24_H_20_P^+^ tetra­phenyl­phospho­nium cations and three aceto­nitrile solvent mol­ecules, one of which is disordered over two sets of sites. The oxabora­phenanthrene moieties of both the B1 and B1*A* anions are again essentially planar (r.m.s. deviations = 0.042 and 0.093 Å, respectively). The bond lengths involving B and O [B1—C1 = 1.603 (2); B1—O1 = 1.466 (2); B1—F1 = 1.412 (2); B1—F2 = 1.411 (2), C11—O1 = 1.3492 (19) Å and B1*A*—C1*A* = 1.601 (2), B1*A*—O1*A* = 1.475 (2); B1*A*—F1*A* = 1.417 (2); B1*A*—F2*A* = 1.415 (2); C11*A*—O1*A* = 1.3435 (19) Å] show some slight variations between (**II**) and (**III**). In (**II**), the B—F bonds are longer than in (**III**), which is presumably due to the F⋯K contacts.

## Supra­molecular features   

**Figure 5:**
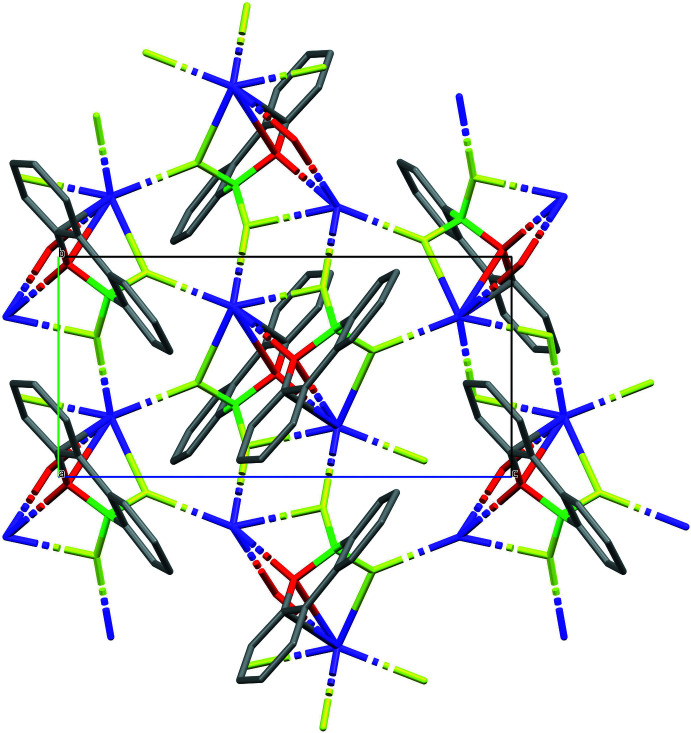
Packing diagram of (**II**) with a view onto the *bc* plane. H atoms are omitted for clarity.

The extended structure of (**II**) features a polymeric network forming layers lying parallel to (001) (Figs. 5 ▸, 6 ▸). K1 is bonded to four F atoms with K—F distances ranging from 2.6132 (9) to 2.7407 (10) Å and two O atoms with K—O distances of 2.7307 (10) and 2.9324 (11) Å. There is one relatively short K⋯C contact [K1⋯C11 = 3.3883 (14)Å] but there are no K⋯π inter­actions.

**Figure 7:**
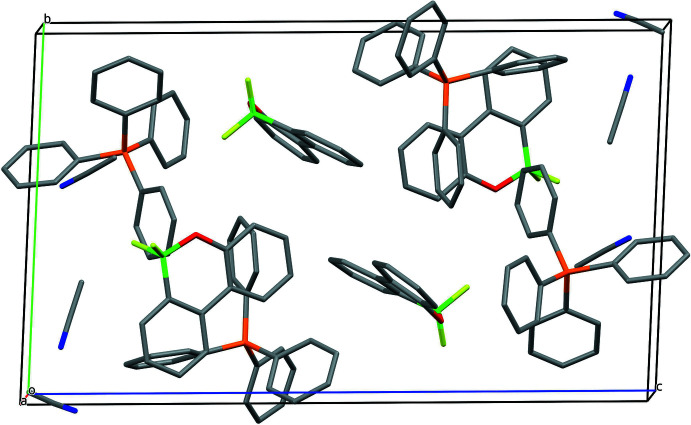
Packing diagram of (**III**) viewed along the *a*-axis direction. H atoms and the minor occupied sites of the disordered aceto­nitrile mol­ecule are omitted for clarity.

In the extended structure of (**III**) (Fig. 7 ▸), the component species are linked by numerous C—H⋯O, C—H⋯N and C—H⋯F inter­actions (Table 1 ▸) into a three-dimensional network.

## Database survey   

There are no structures in the Cambridge Structural Database (version 5.41 of November 2019 plus three updates; Groom *et al.*, 2016 ▸) containing the 6,6′-di­fluoro-6*H*-dibenzo[*c*,*e*][1,2]oxaborinine anion. When the F ligands in the query are changed to any ligand, three structures are found, namely (6*H*-dibenzo[*c*,*e*][1,2]oxadihydridoborato-*O*)(tetra­hydro­furan)(tetra­methyl­ethylenedi­amine)­lithium (CSD refcode CADVIN; Knizek & Nöth, 2000 ▸), [6-phenyl-6-(phen­yl)-6*H*-6-dibenzo[*c*,*e*][1,2]oxaborinine]bis­(tetra­hydro­furan)­lithium benzene solvate (TUZTAL; Budanow *et al.*, 2016 ▸) and (9-*tert*-butyl-9*H*-9-benzo[*c*][1,2]benzoxaborinino[4,3,2-*ij*][2,1]benzoxaborinine)bis­(tetra­hydro­furan)­lithium (9-*tert*-butyl-9*H*-9-benzo[*c*][1,2]benzoxaborinino[4,3,2-*ij*][2,1]benzoxaborinine)tris­(tetra­hydro­furan)­lithium (RUHZUS; Sato *et al.*, 2020 ▸). Since in RUHZUS, the ligands at B are involved in a ring closure, this structure is excluded from the comparison with (**II**) and (**III**).

The bond lengths involving B and O in these structures are: CADVIN: B—C = 1.589, B—O = 1.534, C—O = 1.360 Å; TUZTAL: B—C = 1.611, B—O = 1.543, C—O = 1.358 Å. Whereas the B—C and the O—C bonds are in the same range as in (**II**) and (**III**), the B—O bond is significantly longer than in (**II**) and (**III**). It is notable that the oxabora­phenanthrene moieties in CADVIN and TUZTAL are far more distorted from planarity than in (**II**) and (**III**): the dihedral angles between the aromatic rings are 16.9° in CADVIN and 19.1° in TUZTAL. These dihedral angles are 11.09 (9)° in (**II**) and 4.53 (7) and 9.68 (8)° in (**III**).

## Synthesis and crystallization   

**Synthesis of 9,9-di­fluorido-10,9-oxabora­phenanthrene potassium (II):**

To a solution of 9-hydroxooxabora­phenanthrene (**I**) (1.556 g, 7.94 mmol) in methanol (70 ml) a solution of KHF_2_ (2.669 g, 34.17 mmol) in methanol (30 ml) and water (30 ml) was added at room temperature and the resulting reacting mixture was stirred for 12 h. After removal of all volatiles *in vacuo*, the residue was extracted into aceto­nitrile (30 ml). The colorless suspension was filtered. All volatiles were removed *in vacuo*. The product was obtained as a colorless powder in a yield of 66% (1.34 g, 5.23 mmol). Slow evaporation of a saturated aceto­nitrile solution of (**II)** over *Granopent* led to colorless plates, which were suitable for an investigation by X-ray crystallography.

**Synthesis of 9,9-di­fluorido-10,9-oxabora­phenanthrene tetra­phenyl­phospho­nium (III):**

To a solution of (**II**) (0.511 g, 2.0 mmol) in aceto­nitrile (25 ml) Ph_4_PBr (0.88 g, 2.10 mmol) was added at room temperature and the resulting reacting mixture was stirred for 12 h. The colorless suspension was filtered. After removal of all volatiles, the product was obtained as a colorless powder in a yield of 92% (0.221 g, 0.36 mmol). Slow evaporation of a saturated aceto­nitrile solution of (**III**) over *Granopent* led to colorless blocks, which were suitable for an investigation by X-ray crystallography.

## Refinement   

Crystal data, data collection and structure refinement details are summarized in Table 2 ▸. The H atoms for both structures were refined using a riding model with C—H = 0.95 Å and with *U*_iso_(H) = 1.2*U*_eq_(C) or with C_meth­yl_—H = 0.98 Å and with *U*_iso_(H) = 1.5*U*_eq_(C). The methyl groups were allowed to rotate but not to tip. In (**III**), the C≡N group of one aceto­nitrile mol­ecule is disordered over two sets of sites with a site occupation factor of 0.545 (5) for the major disorder component.

## Supplementary Material

Crystal structure: contains datablock(s) II, III, global. DOI: 10.1107/S2056989020015066/hb7952sup1.cif

Structure factors: contains datablock(s) II. DOI: 10.1107/S2056989020015066/hb7952IIsup2.hkl

Structure factors: contains datablock(s) III. DOI: 10.1107/S2056989020015066/hb7952IIIsup3.hkl

Supporting information file. DOI: 10.1107/S2056989020015066/hb7952IIsup4.cml

Supporting information file. DOI: 10.1107/S2056989020015066/hb7952IIIsup5.cml

CCDC references: 2043934, 2043933

Additional supporting information:  crystallographic information; 3D view; checkCIF report

## Figures and Tables

**Figure 4:**
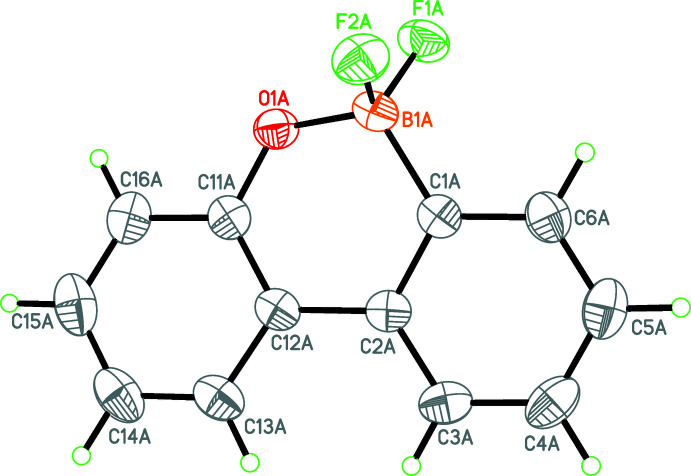
A perspective view of the second of the two anions in the asymmetric unit of (**III**). Displacement ellipsoids are drawn at the 50% probability level.

**Figure 6:**
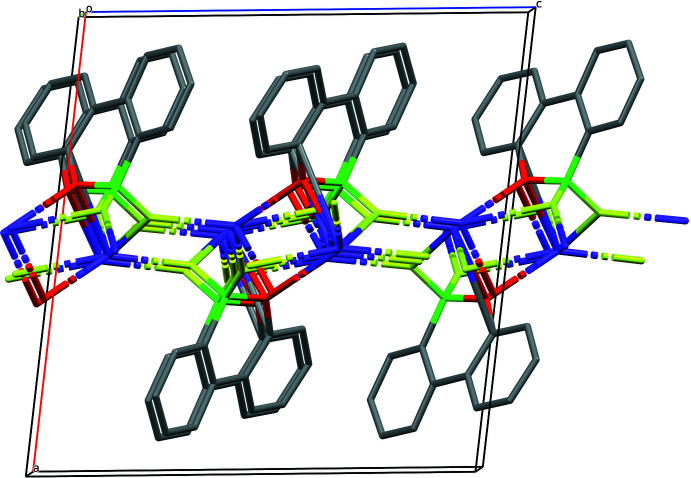
Packing diagram of (**II**) viewed along the *b-*axis direction. H atoms are omitted for clarity.

**Table 1:** Hydrogen-bond geometry (Å, °) for (**III**)

*D*—H⋯*A*	*D*—H	H⋯*A*	*D*⋯*A*	*D*—H⋯*A*
C26*A*—H26*A*⋯O1*A*	0.95	2.58	3.322 (2)	135
C32*A*—H32*A*⋯F2^i^	0.95	2.40	3.069 (2)	127
C42*A*—H42*A*⋯F2^i^	0.95	2.51	3.378 (2)	152
C44*A*—H44*A*⋯F1*A* ^i^	0.95	2.54	3.482 (2)	172
C53—H53⋯O1^i^	0.95	2.59	3.389 (2)	142
C54*A*—H54*A*⋯F2^ii^	0.95	2.55	3.436 (3)	156
C55*A*—H55*A*⋯N81′^i^	0.95	2.52	3.180 (8)	127
C63—H63*A*⋯N61^iii^	0.98	2.58	3.365 (6)	137
C63—H63*C*⋯N71^iv^	0.98	2.47	3.371 (7)	153
C73—H73*A*⋯F1	0.98	2.19	3.106 (4)	156
C73—H73*B*⋯N81^ii^	0.98	2.45	3.313 (10)	146
C83—H83*A*⋯F1	0.98	2.52	3.252 (4)	131

Symmetry codes: (i) 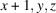; (ii) 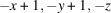; (iii) 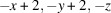; (iv) 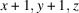.

**Table 2:** Experimental details

	(**II**)	(**III**)
Crystal data		
Chemical formula	K^+^·C_12_H_8_BF_2_O^−^	2C_24_H_20_P^+^·2C_12_H_8_BF_2_O^−^·3C_2_H_3_N
*M*_r_	256.09	1235.89
Crystal system, space group	Monoclinic, *P*2_1_/*c*	Triclinic, *P* 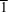
Temperature (K)	173	173
*a*, *b*, *c* (Å)	13.2962 (10), 6.2300 (4), 12.9294 (11)	10.3527 (4), 13.5958 (5), 23.4352 (9)
α, β, γ (°)	90, 97.226 (6), 90	86.218 (3), 78.916 (3), 87.104 (3)
*V* (Å^3^)	1062.50 (14)	3227.6 (2)
*Z*	4	2
Radiation type	Mo *K*α	Mo *K*α
μ (mm^−1^)	0.50	0.13
Crystal size (mm)	0.48 × 0.46 × 0.23	0.42 × 0.38 × 0.29

Data collection		
Diffractometer	Stoe IPDS II two-circle	Stoe IPDS II two-circle
Absorption correction	Multi-scan (*X-AREA*; Stoe & Cie, 2001 ▸)	Multi-scan (*X-AREA*; Stoe & Cie, 2001 ▸)
*T*_min_, *T*_max_	0.794, 0.893	0.947, 0.963
No. of measured, independent and observed [*I* > 2σ(*I*)] reflections	6459, 1980, 1796	47827, 11854, 10863
*R*_int_	0.050	0.040
(sin θ/λ)_max_ (Å^−1^)	0.607	0.604

Refinement		
*R*[*F* ^2^ > 2σ(*F* ^2^)], *wR*(*F* ^2^), *S*	0.030, 0.083, 1.06	0.041, 0.109, 1.05
No. of reflections	1980	11854
No. of parameters	154	842
H-atom treatment	H-atom parameters constrained	H-atom parameters constrained
Δρ_max_, Δρ_min_ (e Å^−3^)	0.26, −0.43	0.48, −0.44

Computer programs: *X-AREA* (Stoe & Cie, 2001 ▸), *SHELXT* (Sheldrick, 2015*a* ▸), *SHELXL* (Sheldrick, 2015*b* ▸), *XP* (Sheldrick, 2015*b* ▸), *Mercury* (Macrae *et al.*, 2020 ▸) and *publCIF* (Westrip, 2010 ▸).

## supplementary crystallographic information

### Potassium 6,6-difluoro-6H-dibenzo[c,e][1,2]oxaborinin-6-ide (II) Crystal data

**Table:**

K^+^·C_12_H_8_BF_2_O^−^	*F*(000) = 520
*M**_r_* = 256.09	*D*_x_ = 1.601 Mg m^−^^3^
Monoclinic, *P*2_1_/*c*	Mo *K*α radiation, λ = 0.71073 Å
*a* = 13.2962 (10) Å	Cell parameters from 6730 reflections
*b* = 6.2300 (4) Å	θ = 3.6–26.0°
*c* = 12.9294 (11) Å	µ = 0.50 mm^−^^1^
β = 97.226 (6)°	*T* = 173 K
*V* = 1062.50 (14) Å^3^	Plate, colourless
*Z* = 4	0.48 × 0.46 × 0.23 mm

### Potassium 6,6-difluoro-6H-dibenzo[c,e][1,2]oxaborinin-6-ide (II) Data collection

**Table:**

STOE IPDS II two-circle diffractometer	1796 reflections with *I* > 2σ(*I*)
ω scans	*R*_int_ = 0.050
Absorption correction: multi-scan (X-Area; Stoe & Cie, 2001)	θ_max_ = 25.6°, θ_min_ = 3.6°
*T*_min_ = 0.794, *T*_max_ = 0.893	*h* = −16→16
6459 measured reflections	*k* = −7→7
1980 independent reflections	*l* = −15→13

### Potassium 6,6-difluoro-6H-dibenzo[c,e][1,2]oxaborinin-6-ide (II) Refinement

**Table:**

Refinement on *F*^2^	Primary atom site location: structure-invariant direct methods
Least-squares matrix: full	Secondary atom site location: difference Fourier map
*R*[*F*^2^ > 2σ(*F*^2^)] = 0.030	Hydrogen site location: inferred from neighbouring sites
*wR*(*F*^2^) = 0.083	H-atom parameters constrained
*S* = 1.06	*w* = 1/[σ^2^(*F*_o_^2^) + (0.0507*P*)^2^ + 0.1783*P*] where *P* = (*F*_o_^2^ + 2*F*_c_^2^)/3
1980 reflections	(Δ/σ)_max_ < 0.001
154 parameters	Δρ_max_ = 0.26 e Å^−^^3^
0 restraints	Δρ_min_ = −0.43 e Å^−^^3^

### Potassium 6,6-difluoro-6H-dibenzo[c,e][1,2]oxaborinin-6-ide (II) Special details

**Table:**

Geometry. All esds (except the esd in the dihedral angle between two l.s. planes) are estimated using the full covariance matrix. The cell esds are taken into account individually in the estimation of esds in distances, angles and torsion angles; correlations between esds in cell parameters are only used when they are defined by crystal symmetry. An approximate (isotropic) treatment of cell esds is used for estimating esds involving l.s. planes.

### Potassium 6,6-difluoro-6H-dibenzo[c,e][1,2]oxaborinin-6-ide (II) Fractional atomic coordinates and isotropic or equivalent isotropic displacement parameters (Å^2^)

**Table:**

	*x*	*y*	*z*	*U*_iso_*/*U*_eq_	
K1	0.47144 (2)	0.77289 (5)	0.38440 (2)	0.02252 (13)	
B1	0.61842 (12)	0.3281 (3)	0.38931 (12)	0.0182 (3)	
O1	0.62336 (7)	0.46771 (16)	0.48017 (7)	0.0201 (2)	
F1	0.55034 (6)	0.42677 (15)	0.30658 (6)	0.0258 (2)	
F2	0.56553 (6)	0.13979 (14)	0.41608 (7)	0.0246 (2)	
C1	0.72587 (11)	0.2796 (2)	0.35252 (11)	0.0186 (3)	
C2	0.80729 (11)	0.4170 (2)	0.38571 (11)	0.0195 (3)	
C3	0.90255 (11)	0.3756 (3)	0.35387 (12)	0.0249 (3)	
H3	0.958382	0.465504	0.377711	0.030*	
C4	0.91617 (12)	0.2057 (3)	0.28830 (13)	0.0279 (4)	
H4	0.980868	0.181458	0.266586	0.034*	
C5	0.83640 (12)	0.0709 (3)	0.25411 (12)	0.0271 (3)	
H5	0.845540	−0.044942	0.208452	0.033*	
C6	0.74217 (12)	0.1076 (3)	0.28767 (11)	0.0226 (3)	
H6	0.687666	0.012800	0.265759	0.027*	
C11	0.70096 (11)	0.6112 (2)	0.50227 (11)	0.0177 (3)	
C12	0.79123 (11)	0.5970 (2)	0.45628 (11)	0.0194 (3)	
C13	0.86343 (12)	0.7582 (2)	0.48232 (13)	0.0253 (3)	
H13	0.924129	0.756567	0.450636	0.030*	
C14	0.84952 (12)	0.9195 (3)	0.55239 (13)	0.0285 (4)	
H14	0.899937	1.026782	0.567981	0.034*	
C15	0.76149 (12)	0.9244 (2)	0.60003 (12)	0.0263 (3)	
H15	0.752271	1.032398	0.649848	0.032*	
C16	0.68731 (12)	0.7707 (2)	0.57435 (12)	0.0221 (3)	
H16	0.626722	0.774242	0.606210	0.027*	

### Potassium 6,6-difluoro-6H-dibenzo[c,e][1,2]oxaborinin-6-ide (II) Atomic displacement parameters (Å^2^)

**Table:**

	*U*^11^	*U*^22^	*U*^33^	*U*^12^	*U*^13^	*U*^23^
K1	0.0320 (2)	0.01629 (19)	0.01999 (19)	−0.00165 (13)	0.00615 (13)	0.00069 (11)
B1	0.0191 (7)	0.0158 (7)	0.0197 (7)	−0.0023 (6)	0.0025 (6)	−0.0009 (6)
O1	0.0187 (5)	0.0208 (5)	0.0215 (5)	−0.0053 (4)	0.0048 (4)	−0.0038 (4)
F1	0.0269 (5)	0.0275 (5)	0.0218 (4)	0.0072 (4)	−0.0016 (3)	−0.0030 (3)
F2	0.0244 (4)	0.0189 (4)	0.0317 (5)	−0.0070 (4)	0.0085 (3)	−0.0034 (4)
C1	0.0210 (7)	0.0182 (7)	0.0166 (6)	0.0005 (6)	0.0028 (5)	0.0031 (5)
C2	0.0200 (7)	0.0206 (7)	0.0181 (6)	0.0001 (6)	0.0032 (5)	0.0038 (6)
C3	0.0197 (7)	0.0289 (8)	0.0266 (7)	−0.0015 (6)	0.0052 (6)	0.0014 (6)
C4	0.0222 (8)	0.0355 (9)	0.0275 (8)	0.0053 (7)	0.0084 (6)	0.0002 (7)
C5	0.0310 (8)	0.0262 (8)	0.0249 (7)	0.0066 (7)	0.0062 (6)	−0.0024 (6)
C6	0.0235 (7)	0.0212 (7)	0.0231 (7)	−0.0004 (6)	0.0029 (6)	−0.0001 (6)
C11	0.0174 (6)	0.0159 (6)	0.0188 (6)	−0.0017 (6)	−0.0013 (5)	0.0022 (5)
C12	0.0201 (7)	0.0191 (7)	0.0184 (6)	−0.0013 (6)	0.0002 (5)	0.0031 (5)
C13	0.0215 (7)	0.0253 (8)	0.0290 (8)	−0.0054 (6)	0.0028 (6)	0.0018 (6)
C14	0.0285 (8)	0.0217 (8)	0.0335 (8)	−0.0077 (7)	−0.0028 (6)	−0.0018 (6)
C15	0.0295 (8)	0.0189 (7)	0.0286 (8)	0.0013 (6)	−0.0038 (6)	−0.0037 (6)
C16	0.0224 (7)	0.0211 (7)	0.0222 (7)	0.0031 (6)	0.0002 (6)	−0.0008 (6)

### Potassium 6,6-difluoro-6H-dibenzo[c,e][1,2]oxaborinin-6-ide (II) Geometric parameters (Å, º)

**Table:**

K1—F2^i^	2.6132 (9)	C2—C3	1.404 (2)
K1—F1^ii^	2.6307 (9)	C2—C12	1.478 (2)
K1—F1	2.6511 (9)	C3—C4	1.382 (2)
K1—O1^iii^	2.7307 (10)	C3—H3	0.9500
K1—F2^iii^	2.7407 (10)	C4—C5	1.381 (2)
K1—O1	2.9324 (11)	C4—H4	0.9500
K1—B1^iii^	3.3560 (16)	C5—C6	1.395 (2)
K1—B1	3.3867 (17)	C5—H5	0.9500
K1—C11	3.3883 (14)	C6—H6	0.9500
K1—C6^ii^	3.5338 (16)	C11—C16	1.390 (2)
K1—C16	3.5351 (15)	C11—C12	1.408 (2)
K1—K1^iv^	4.1169 (6)	C12—C13	1.401 (2)
B1—F2	1.4325 (18)	C13—C14	1.381 (2)
B1—F1	1.4483 (18)	C13—H13	0.9500
B1—O1	1.4564 (19)	C14—C15	1.390 (2)
B1—C1	1.591 (2)	C14—H14	0.9500
O1—C11	1.3678 (17)	C15—C16	1.385 (2)
C1—C6	1.395 (2)	C15—H15	0.9500
C1—C2	1.404 (2)	C16—H16	0.9500
			
F2^i^—K1—F1^ii^	79.77 (3)	F2—B1—C1	113.79 (12)
F2^i^—K1—F1	124.50 (3)	F1—B1—C1	111.18 (12)
F1^ii^—K1—F1	86.689 (19)	O1—B1—C1	113.80 (12)
F2^i^—K1—O1^iii^	129.06 (3)	F2—B1—K1^iii^	52.87 (6)
F1^ii^—K1—O1^iii^	142.82 (3)	F1—B1—K1^iii^	117.74 (9)
F1—K1—O1^iii^	91.92 (3)	O1—B1—K1^iii^	52.67 (6)
F2^i^—K1—F2^iii^	79.51 (3)	C1—B1—K1^iii^	131.05 (9)
F1^ii^—K1—F2^iii^	143.09 (3)	F2—B1—K1	112.03 (9)
F1—K1—F2^iii^	130.17 (3)	F1—B1—K1	48.20 (6)
O1^iii^—K1—F2^iii^	49.72 (3)	O1—B1—K1	59.60 (7)
F2^i^—K1—O1	102.13 (3)	C1—B1—K1	133.45 (9)
F1^ii^—K1—O1	128.41 (3)	K1^iii^—B1—K1	84.37 (4)
F1—K1—O1	49.34 (3)	C11—O1—B1	121.00 (11)
O1^iii^—K1—O1	73.88 (3)	C11—O1—K1^iii^	128.44 (8)
F2^iii^—K1—O1	85.80 (3)	B1—O1—K1^iii^	102.23 (8)
F2^i^—K1—B1^iii^	104.08 (3)	C11—O1—K1	97.24 (8)
F1^ii^—K1—B1^iii^	151.33 (4)	B1—O1—K1	95.03 (8)
F1—K1—B1^iii^	112.31 (4)	K1^iii^—O1—K1	106.12 (3)
O1^iii^—K1—B1^iii^	25.09 (3)	B1—F1—K1^v^	121.56 (8)
F2^iii^—K1—B1^iii^	24.63 (3)	B1—F1—K1	107.77 (8)
O1—K1—B1^iii^	79.17 (3)	K1^v^—F1—K1	130.59 (3)
F2^i^—K1—B1	116.68 (4)	B1—F2—K1^vi^	156.70 (8)
F1^ii^—K1—B1	108.11 (4)	B1—F2—K1^iii^	102.50 (8)
F1—K1—B1	24.03 (3)	K1^vi^—F2—K1^iii^	100.49 (3)
O1^iii^—K1—B1	81.41 (4)	C6—C1—C2	118.68 (13)
F2^iii^—K1—B1	108.43 (3)	C6—C1—B1	122.63 (13)
O1—K1—B1	25.37 (3)	C2—C1—B1	118.69 (13)
B1^iii^—K1—B1	95.63 (4)	C1—C2—C3	119.20 (14)
F2^i^—K1—C11	78.65 (3)	C1—C2—C12	119.03 (13)
F1^ii^—K1—C11	121.14 (3)	C3—C2—C12	121.73 (14)
F1—K1—C11	63.37 (3)	C4—C3—C2	120.87 (15)
O1^iii^—K1—C11	90.61 (3)	C4—C3—H3	119.6
F2^iii^—K1—C11	83.90 (3)	C2—C3—H3	119.6
O1—K1—C11	23.61 (3)	C5—C4—C3	120.48 (14)
B1^iii^—K1—C11	87.21 (4)	C5—C4—H4	119.8
B1—K1—C11	42.55 (4)	C3—C4—H4	119.8
F2^i^—K1—C6^ii^	133.20 (3)	C4—C5—C6	118.98 (14)
F1^ii^—K1—C6^ii^	61.99 (3)	C4—C5—H5	120.5
F1—K1—C6^ii^	81.32 (3)	C6—C5—H5	120.5
O1^iii^—K1—C6^ii^	81.05 (3)	C1—C6—C5	121.75 (15)
F2^iii^—K1—C6^ii^	115.17 (3)	C1—C6—K1^v^	88.92 (9)
O1—K1—C6^ii^	122.23 (3)	C5—C6—K1^v^	122.27 (10)
B1^iii^—K1—C6^ii^	98.52 (4)	C1—C6—H6	119.1
B1—K1—C6^ii^	100.93 (4)	C5—C6—H6	119.1
C11—K1—C6^ii^	143.48 (4)	K1^v^—C6—H6	58.0
F2^i^—K1—C16	64.13 (3)	O1—C11—C16	116.65 (13)
F1^ii^—K1—C16	129.60 (3)	O1—C11—C12	122.12 (13)
F1—K1—C16	86.27 (3)	C16—C11—C12	121.22 (14)
O1^iii^—K1—C16	87.27 (3)	O1—C11—K1	59.16 (6)
F2^iii^—K1—C16	64.72 (3)	C16—C11—K1	84.42 (9)
O1—K1—C16	41.25 (3)	C12—C11—K1	126.61 (9)
B1^iii^—K1—C16	74.96 (4)	C13—C12—C11	116.64 (14)
B1—K1—C16	64.38 (4)	C13—C12—C2	123.00 (13)
C11—K1—C16	23.03 (3)	C11—C12—C2	120.35 (13)
C6^ii^—K1—C16	162.61 (4)	C14—C13—C12	122.36 (15)
F2^i^—K1—K1^iv^	40.89 (2)	C14—C13—H13	118.8
F1^ii^—K1—K1^iv^	114.80 (2)	C12—C13—H13	118.8
F1—K1—K1^iv^	142.09 (2)	C13—C14—C15	119.76 (15)
O1^iii^—K1—K1^iv^	88.26 (2)	C13—C14—H14	120.1
F2^iii^—K1—K1^iv^	38.619 (19)	C15—C14—H14	120.1
O1—K1—K1^iv^	94.86 (2)	C16—C15—C14	119.52 (14)
B1^iii^—K1—K1^iv^	63.21 (3)	C16—C15—H15	120.2
B1—K1—K1^iv^	119.70 (3)	C14—C15—H15	120.2
C11—K1—K1^iv^	78.72 (3)	C15—C16—C11	120.40 (15)
C6^ii^—K1—K1^iv^	135.87 (3)	C15—C16—K1	130.95 (10)
C16—K1—K1^iv^	55.86 (3)	C11—C16—K1	72.54 (8)
F2—B1—F1	104.23 (11)	C15—C16—H16	119.8
F2—B1—O1	105.54 (11)	C11—C16—H16	119.8
F1—B1—O1	107.63 (12)	K1—C16—H16	69.1
			
F2—B1—O1—C11	151.69 (11)	B1—C1—C2—C3	179.90 (13)
F1—B1—O1—C11	−97.45 (14)	C6—C1—C2—C12	−178.70 (13)
C1—B1—O1—C11	26.22 (18)	B1—C1—C2—C12	2.01 (19)
K1^iii^—B1—O1—C11	150.72 (13)	C1—C2—C3—C4	1.8 (2)
K1—B1—O1—C11	−101.56 (11)	C12—C2—C3—C4	179.63 (14)
F2—B1—O1—K1^iii^	0.97 (11)	C2—C3—C4—C5	−1.1 (2)
F1—B1—O1—K1^iii^	111.83 (10)	C3—C4—C5—C6	−0.7 (2)
C1—B1—O1—K1^iii^	−124.50 (10)	C2—C1—C6—C5	−0.9 (2)
K1—B1—O1—K1^iii^	107.72 (5)	B1—C1—C6—C5	178.34 (14)
F2—B1—O1—K1	−106.75 (9)	C2—C1—C6—K1^v^	−128.97 (12)
F1—B1—O1—K1	4.11 (11)	B1—C1—C6—K1^v^	50.29 (13)
C1—B1—O1—K1	127.79 (11)	C4—C5—C6—C1	1.7 (2)
K1^iii^—B1—O1—K1	−107.72 (5)	C4—C5—C6—K1^v^	113.06 (15)
F2—B1—F1—K1^v^	−76.01 (12)	B1—O1—C11—C16	165.10 (13)
O1—B1—F1—K1^v^	172.24 (7)	K1^iii^—O1—C11—C16	−52.50 (16)
C1—B1—F1—K1^v^	46.99 (14)	K1—O1—C11—C16	64.76 (12)
K1^iii^—B1—F1—K1^v^	−131.24 (6)	B1—O1—C11—C12	−16.10 (19)
K1—B1—F1—K1^v^	177.00 (10)	K1^iii^—O1—C11—C12	126.30 (11)
F2—B1—F1—K1	106.99 (9)	K1—O1—C11—C12	−116.44 (12)
O1—B1—F1—K1	−4.76 (12)	B1—O1—C11—K1	100.34 (12)
C1—B1—F1—K1	−130.01 (10)	K1^iii^—O1—C11—K1	−117.26 (9)
K1^iii^—B1—F1—K1	51.76 (9)	O1—C11—C12—C13	177.69 (13)
F1—B1—F2—K1^vi^	56.3 (3)	C16—C11—C12—C13	−3.6 (2)
O1—B1—F2—K1^vi^	169.55 (14)	K1—C11—C12—C13	104.41 (13)
C1—B1—F2—K1^vi^	−65.0 (3)	O1—C11—C12—C2	−3.2 (2)
K1^iii^—B1—F2—K1^vi^	170.5 (2)	C16—C11—C12—C2	175.51 (13)
K1—B1—F2—K1^vi^	106.56 (19)	K1—C11—C12—C2	−76.51 (16)
F1—B1—F2—K1^iii^	−114.22 (9)	C1—C2—C12—C13	−171.25 (13)
O1—B1—F2—K1^iii^	−0.96 (11)	C3—C2—C12—C13	10.9 (2)
C1—B1—F2—K1^iii^	124.51 (10)	C1—C2—C12—C11	9.7 (2)
K1—B1—F2—K1^iii^	−63.96 (7)	C3—C2—C12—C11	−168.10 (13)
F2—B1—C1—C6	40.77 (19)	C11—C12—C13—C14	2.3 (2)
F1—B1—C1—C6	−76.55 (17)	C2—C12—C13—C14	−176.70 (14)
O1—B1—C1—C6	161.72 (13)	C12—C13—C14—C15	0.3 (2)
K1^iii^—B1—C1—C6	101.37 (15)	C13—C14—C15—C16	−1.9 (2)
K1—B1—C1—C6	−128.40 (13)	C14—C15—C16—C11	0.6 (2)
F2—B1—C1—C2	−139.97 (13)	C14—C15—C16—K1	−92.15 (17)
F1—B1—C1—C2	102.71 (15)	O1—C11—C16—C15	−179.02 (13)
O1—B1—C1—C2	−19.02 (18)	C12—C11—C16—C15	2.2 (2)
K1^iii^—B1—C1—C2	−79.37 (16)	K1—C11—C16—C15	−127.73 (13)
K1—B1—C1—C2	50.86 (18)	O1—C11—C16—K1	−51.29 (10)
C6—C1—C2—C3	−0.8 (2)	C12—C11—C16—K1	129.90 (13)

Symmetry codes: (i) *x*, *y*+1, *z*; (ii) −*x*+1, *y*+1/2, −*z*+1/2; (iii) −*x*+1, −*y*+1, −*z*+1; (iv) −*x*+1, −*y*+2, −*z*+1; (v) −*x*+1, *y*−1/2, −*z*+1/2; (vi) *x*, *y*−1, *z*.

### Bis(tetraphenylphosphonium) 6,6-difluoro-6H-dibenzo[c,e][1,2]oxaborinin-6-ide acetonitrile trisolvate (III) Crystal data

**Table:**

2C_24_H_20_P^+^·2C_12_H_8_BF_2_O^−^·3C_2_H_3_N	*Z* = 2
*M**_r_* = 1235.89	*F*(000) = 1292
Triclinic, *P*1	*D*_x_ = 1.272 Mg m^−^^3^
*a* = 10.3527 (4) Å	Mo *K*α radiation, λ = 0.71073 Å
*b* = 13.5958 (5) Å	Cell parameters from 61056 reflections
*c* = 23.4352 (9) Å	θ = 2.0–25.9°
α = 86.218 (3)°	µ = 0.13 mm^−^^1^
β = 78.916 (3)°	*T* = 173 K
γ = 87.104 (3)°	Block, colourless
*V* = 3227.6 (2) Å^3^	0.42 × 0.38 × 0.29 mm

### Bis(tetraphenylphosphonium) 6,6-difluoro-6H-dibenzo[c,e][1,2]oxaborinin-6-ide acetonitrile trisolvate (III) Data collection

**Table:**

STOE IPDS II two-circle diffractometer	10863 reflections with *I* > 2σ(*I*)
ω scans	*R*_int_ = 0.040
Absorption correction: multi-scan (X-Area; Stoe & Cie, 2001)	θ_max_ = 25.4°, θ_min_ = 2.0°
*T*_min_ = 0.947, *T*_max_ = 0.963	*h* = −12→12
47827 measured reflections	*k* = −16→16
11854 independent reflections	*l* = −27→28

### Bis(tetraphenylphosphonium) 6,6-difluoro-6H-dibenzo[c,e][1,2]oxaborinin-6-ide acetonitrile trisolvate (III) Refinement

**Table:**

Refinement on *F*^2^	Primary atom site location: structure-invariant direct methods
Least-squares matrix: full	Hydrogen site location: inferred from neighbouring sites
*R*[*F*^2^ > 2σ(*F*^2^)] = 0.041	H-atom parameters constrained
*wR*(*F*^2^) = 0.109	*w* = 1/[σ^2^(*F*_o_^2^) + (0.0516*P*)^2^ + 1.4271*P*] where *P* = (*F*_o_^2^ + 2*F*_c_^2^)/3
*S* = 1.05	(Δ/σ)_max_ = 0.001
11854 reflections	Δρ_max_ = 0.48 e Å^−^^3^
842 parameters	Δρ_min_ = −0.44 e Å^−^^3^
0 restraints	

### Bis(tetraphenylphosphonium) 6,6-difluoro-6H-dibenzo[c,e][1,2]oxaborinin-6-ide acetonitrile trisolvate (III) Special details

**Table:**

Geometry. All esds (except the esd in the dihedral angle between two l.s. planes) are estimated using the full covariance matrix. The cell esds are taken into account individually in the estimation of esds in distances, angles and torsion angles; correlations between esds in cell parameters are only used when they are defined by crystal symmetry. An approximate (isotropic) treatment of cell esds is used for estimating esds involving l.s. planes.

### Bis(tetraphenylphosphonium) 6,6-difluoro-6H-dibenzo[c,e][1,2]oxaborinin-6-ide acetonitrile trisolvate (III) Fractional atomic coordinates and isotropic or equivalent isotropic displacement parameters (Å^2^)

**Table:**

	*x*	*y*	*z*	*U*_iso_*/*U*_eq_	Occ. (<1)
O1	0.12452 (13)	0.43049 (8)	0.25804 (5)	0.0398 (3)	
B1	0.12323 (19)	0.37459 (13)	0.20674 (8)	0.0328 (4)	
F1	0.22126 (13)	0.41208 (8)	0.16118 (5)	0.0551 (3)	
F2	0.00205 (12)	0.39835 (8)	0.18908 (5)	0.0531 (3)	
C1	0.14294 (14)	0.25805 (11)	0.21957 (7)	0.0292 (3)	
C2	0.17145 (14)	0.21953 (11)	0.27317 (7)	0.0272 (3)	
C3	0.18519 (15)	0.11698 (11)	0.28362 (7)	0.0315 (3)	
H3	0.204651	0.091107	0.319803	0.038*	
C4	0.17073 (16)	0.05318 (12)	0.24187 (8)	0.0354 (4)	
H4	0.179876	−0.016049	0.249542	0.043*	
C5	0.14293 (16)	0.09020 (12)	0.18890 (8)	0.0371 (4)	
H5	0.133030	0.046587	0.160114	0.045*	
C6	0.12967 (16)	0.19123 (12)	0.17818 (7)	0.0353 (4)	
H6	0.111004	0.216017	0.141658	0.042*	
C11	0.15851 (15)	0.39085 (11)	0.30763 (7)	0.0312 (3)	
C12	0.18343 (14)	0.28890 (11)	0.31781 (7)	0.0268 (3)	
C13	0.21755 (15)	0.25770 (12)	0.37135 (7)	0.0311 (3)	
H13	0.235772	0.189468	0.379119	0.037*	
C14	0.22553 (16)	0.32294 (13)	0.41318 (7)	0.0345 (3)	
H14	0.247785	0.299361	0.449264	0.041*	
C15	0.20102 (17)	0.42267 (13)	0.40237 (7)	0.0373 (4)	
H15	0.206986	0.467925	0.430872	0.045*	
C16	0.16792 (18)	0.45616 (12)	0.35008 (8)	0.0381 (4)	
H16	0.151223	0.524744	0.342801	0.046*	
P1	0.67556 (4)	0.15494 (3)	0.35037 (2)	0.02367 (9)	
C21	0.67013 (15)	0.12421 (10)	0.27748 (6)	0.0272 (3)	
C22	0.78659 (17)	0.10811 (12)	0.23759 (7)	0.0356 (4)	
H22	0.869235	0.106773	0.249633	0.043*	
C23	0.7804 (2)	0.09407 (14)	0.18009 (8)	0.0459 (4)	
H23	0.859167	0.082089	0.152714	0.055*	
C24	0.6604 (2)	0.09741 (15)	0.16243 (8)	0.0481 (5)	
H24	0.657115	0.086850	0.123079	0.058*	
C25	0.54518 (19)	0.11599 (13)	0.20154 (8)	0.0411 (4)	
H25	0.463168	0.119617	0.188864	0.049*	
C26	0.54918 (16)	0.12932 (11)	0.25924 (7)	0.0322 (3)	
H26	0.470016	0.141883	0.286267	0.039*	
C31	0.53405 (14)	0.10965 (10)	0.39992 (6)	0.0248 (3)	
C32	0.47498 (15)	0.02422 (11)	0.38891 (7)	0.0281 (3)	
H32	0.505119	−0.007380	0.353556	0.034*	
C33	0.37240 (15)	−0.01386 (11)	0.42989 (7)	0.0318 (3)	
H33	0.331854	−0.071696	0.422591	0.038*	
C34	0.32868 (15)	0.03207 (11)	0.48147 (7)	0.0322 (3)	
H34	0.256819	0.006415	0.508956	0.039*	
C35	0.38893 (15)	0.11528 (11)	0.49337 (7)	0.0309 (3)	
H35	0.359439	0.145623	0.529190	0.037*	
C36	0.49214 (15)	0.15399 (11)	0.45292 (7)	0.0284 (3)	
H36	0.534334	0.210452	0.461115	0.034*	
C41	0.81841 (14)	0.09717 (11)	0.37314 (6)	0.0266 (3)	
C42	0.85557 (16)	0.00071 (11)	0.35834 (7)	0.0335 (3)	
H42	0.811375	−0.031069	0.333132	0.040*	
C43	0.95702 (17)	−0.04846 (13)	0.38051 (8)	0.0400 (4)	
H43	0.983616	−0.113748	0.369988	0.048*	
C44	1.01967 (16)	−0.00275 (13)	0.41791 (8)	0.0402 (4)	
H44	1.089368	−0.036748	0.433015	0.048*	
C45	0.98160 (16)	0.09215 (13)	0.43351 (8)	0.0379 (4)	
H45	1.024512	0.122775	0.459638	0.046*	
C46	0.88125 (15)	0.14277 (12)	0.41124 (7)	0.0314 (3)	
H46	0.855390	0.208128	0.421831	0.038*	
C51	0.68050 (15)	0.28636 (10)	0.34950 (6)	0.0258 (3)	
C52	0.79962 (15)	0.33163 (11)	0.32810 (7)	0.0309 (3)	
H52	0.878918	0.292910	0.318517	0.037*	
C53	0.80107 (17)	0.43362 (12)	0.32097 (7)	0.0351 (4)	
H53	0.881769	0.465079	0.306699	0.042*	
C54	0.68499 (17)	0.48980 (11)	0.33462 (7)	0.0346 (4)	
H54	0.686732	0.559677	0.329805	0.041*	
C55	0.56697 (17)	0.44518 (12)	0.35513 (7)	0.0345 (3)	
H55	0.487928	0.484344	0.364219	0.041*	
C56	0.56353 (15)	0.34322 (11)	0.36250 (7)	0.0301 (3)	
H56	0.482246	0.312297	0.376277	0.036*	
O1A	0.46591 (11)	0.79394 (8)	0.34012 (5)	0.0348 (2)	
B1A	0.32951 (18)	0.76328 (13)	0.34453 (8)	0.0312 (4)	
F1A	0.25501 (10)	0.84978 (7)	0.33229 (5)	0.0436 (2)	
F2A	0.32692 (11)	0.69946 (7)	0.29946 (4)	0.0425 (2)	
C1A	0.27336 (15)	0.71222 (11)	0.40733 (7)	0.0299 (3)	
C2A	0.36027 (16)	0.67457 (11)	0.44359 (7)	0.0289 (3)	
C3A	0.30921 (18)	0.62809 (12)	0.49817 (7)	0.0364 (4)	
H3A	0.367670	0.602655	0.522687	0.044*	
C4A	0.1751 (2)	0.61904 (13)	0.51648 (8)	0.0432 (4)	
H4A	0.141895	0.587255	0.553332	0.052*	
C5A	0.08914 (19)	0.65624 (14)	0.48120 (9)	0.0457 (4)	
H5A	−0.003151	0.650298	0.493725	0.055*	
C6A	0.13839 (17)	0.70203 (13)	0.42769 (8)	0.0390 (4)	
H6A	0.078495	0.727520	0.403857	0.047*	
C11A	0.54740 (16)	0.74683 (11)	0.37201 (7)	0.0307 (3)	
C12A	0.50301 (16)	0.68660 (11)	0.42267 (7)	0.0296 (3)	
C13A	0.59866 (18)	0.64043 (12)	0.45160 (8)	0.0376 (4)	
H13A	0.570852	0.598894	0.485508	0.045*	
C14A	0.73140 (18)	0.65331 (14)	0.43247 (9)	0.0452 (4)	
H14A	0.793671	0.620652	0.452860	0.054*	
C15A	0.77353 (18)	0.71413 (14)	0.38338 (9)	0.0463 (5)	
H15A	0.864899	0.723935	0.370258	0.056*	
C16A	0.68276 (17)	0.76060 (13)	0.35343 (8)	0.0388 (4)	
H16A	0.712373	0.802395	0.319832	0.047*	
P1A	0.74154 (4)	0.67262 (3)	0.14593 (2)	0.02748 (10)	
C21A	0.61990 (15)	0.77059 (11)	0.14360 (7)	0.0290 (3)	
C22A	0.58843 (18)	0.80677 (13)	0.09096 (8)	0.0389 (4)	
H22A	0.633680	0.781568	0.055405	0.047*	
C23A	0.4905 (2)	0.87989 (15)	0.09080 (9)	0.0495 (5)	
H23A	0.469425	0.905880	0.054929	0.059*	
C24A	0.42331 (19)	0.91521 (14)	0.14263 (9)	0.0466 (4)	
H24A	0.355582	0.964914	0.142184	0.056*	
C25A	0.45370 (18)	0.87892 (12)	0.19509 (8)	0.0394 (4)	
H25A	0.406434	0.903138	0.230570	0.047*	
C26A	0.55279 (16)	0.80746 (11)	0.19586 (7)	0.0334 (3)	
H26A	0.575369	0.783373	0.231790	0.040*	
C31A	0.66018 (16)	0.56238 (11)	0.17613 (7)	0.0322 (3)	
C32A	0.72734 (19)	0.47093 (12)	0.16976 (9)	0.0419 (4)	
H32A	0.816655	0.466797	0.150269	0.050*	
C33A	0.6618 (2)	0.38587 (13)	0.19234 (10)	0.0514 (5)	
H33A	0.707222	0.323427	0.188851	0.062*	
C34A	0.5318 (2)	0.39153 (14)	0.21965 (9)	0.0518 (5)	
H34A	0.487228	0.332857	0.233800	0.062*	
C35A	0.4661 (2)	0.48134 (14)	0.22657 (9)	0.0487 (5)	
H35A	0.376676	0.484745	0.245929	0.058*	
C36A	0.52989 (18)	0.56706 (13)	0.20543 (8)	0.0388 (4)	
H36A	0.484588	0.629246	0.210937	0.047*	
C41A	0.85613 (15)	0.70925 (11)	0.18844 (7)	0.0294 (3)	
C42A	0.93100 (18)	0.63853 (12)	0.21477 (8)	0.0374 (4)	
H42A	0.917951	0.570346	0.212493	0.045*	
C43A	1.0246 (2)	0.66824 (13)	0.24431 (9)	0.0457 (4)	
H43A	1.075962	0.620113	0.262275	0.055*	
C44A	1.04410 (19)	0.76758 (13)	0.24791 (9)	0.0437 (4)	
H44A	1.108785	0.787400	0.268126	0.052*	
C45A	0.96920 (18)	0.83766 (13)	0.22203 (8)	0.0417 (4)	
H45A	0.981968	0.905736	0.224882	0.050*	
C46A	0.87565 (17)	0.80948 (12)	0.19197 (8)	0.0353 (4)	
H46A	0.825052	0.857948	0.173851	0.042*	
C51A	0.82688 (17)	0.65247 (13)	0.07317 (7)	0.0368 (4)	
C52A	0.7733 (2)	0.59274 (16)	0.03807 (9)	0.0542 (5)	
H52A	0.695692	0.558136	0.053530	0.065*	
C53A	0.8346 (3)	0.5845 (2)	−0.01946 (10)	0.0737 (8)	
H53A	0.798253	0.544150	−0.043517	0.088*	
C54A	0.9469 (3)	0.6338 (2)	−0.04215 (10)	0.0799 (9)	
H54A	0.987884	0.627627	−0.081730	0.096*	
C55A	1.0004 (3)	0.6921 (2)	−0.00771 (11)	0.0783 (8)	
H55A	1.078704	0.725655	−0.023523	0.094*	
C56A	0.9404 (2)	0.70232 (17)	0.05041 (9)	0.0543 (5)	
H56A	0.977155	0.743069	0.074091	0.065*	
N61	1.0162 (5)	0.9547 (3)	0.07570 (13)	0.1405 (15)	
C62	1.1110 (4)	0.9774 (3)	0.04320 (13)	0.0991 (11)	
C63	1.2218 (4)	1.0112 (5)	0.0041 (2)	0.156 (2)	
H63A	1.195474	1.037192	−0.032195	0.235*	
H63B	1.286666	0.956532	−0.004253	0.235*	
H63C	1.260647	1.063580	0.021245	0.235*	
N71	0.4432 (5)	0.1390 (3)	0.05427 (14)	0.1466 (16)	
C72	0.4540 (4)	0.2169 (3)	0.06671 (11)	0.0886 (9)	
C73	0.4630 (3)	0.3133 (3)	0.08298 (14)	0.0933 (10)	
H73A	0.390831	0.327689	0.115388	0.140*	
H73B	0.457012	0.359912	0.049792	0.140*	
H73C	0.547505	0.319626	0.095136	0.140*	
N81	0.4309 (7)	0.5722 (6)	0.0455 (4)	0.143 (3)	0.545 (5)
C82	0.3563 (6)	0.6043 (5)	0.0833 (4)	0.098 (2)	0.545 (5)
N81'	0.2713 (7)	0.7449 (6)	0.0256 (4)	0.118 (3)	0.455 (5)
C82'	0.2828 (6)	0.6973 (5)	0.0668 (4)	0.0790 (19)	0.455 (5)
C83	0.2753 (5)	0.6434 (3)	0.1246 (2)	0.1204 (14)	
H83A	0.255574	0.595002	0.157706	0.181*	0.545 (5)
H83B	0.193732	0.664258	0.111112	0.181*	0.545 (5)
H83C	0.314423	0.700794	0.136783	0.181*	0.545 (5)
H83D	0.234341	0.580029	0.124309	0.181*	0.455 (5)
H83E	0.222277	0.682864	0.154807	0.181*	0.455 (5)
H83F	0.364246	0.631736	0.132872	0.181*	0.455 (5)

### Bis(tetraphenylphosphonium) 6,6-difluoro-6H-dibenzo[c,e][1,2]oxaborinin-6-ide acetonitrile trisolvate (III) Atomic displacement parameters (Å^2^)

**Table:**

	*U*^11^	*U*^22^	*U*^33^	*U*^12^	*U*^13^	*U*^23^
O1	0.0612 (8)	0.0272 (6)	0.0336 (6)	0.0045 (5)	−0.0171 (6)	−0.0024 (5)
B1	0.0377 (9)	0.0315 (9)	0.0300 (9)	0.0016 (7)	−0.0090 (7)	−0.0014 (7)
F1	0.0722 (8)	0.0427 (6)	0.0432 (6)	−0.0100 (5)	0.0072 (5)	0.0038 (5)
F2	0.0583 (7)	0.0446 (6)	0.0648 (7)	0.0157 (5)	−0.0346 (6)	−0.0109 (5)
C1	0.0244 (7)	0.0316 (8)	0.0313 (8)	−0.0013 (6)	−0.0040 (6)	−0.0041 (6)
C2	0.0209 (7)	0.0284 (7)	0.0313 (8)	−0.0018 (5)	−0.0022 (6)	−0.0027 (6)
C3	0.0286 (8)	0.0294 (8)	0.0360 (8)	−0.0016 (6)	−0.0050 (6)	−0.0010 (6)
C4	0.0305 (8)	0.0279 (8)	0.0471 (10)	−0.0010 (6)	−0.0040 (7)	−0.0063 (7)
C5	0.0346 (8)	0.0357 (9)	0.0427 (10)	−0.0004 (7)	−0.0076 (7)	−0.0146 (7)
C6	0.0351 (8)	0.0387 (9)	0.0334 (9)	0.0001 (7)	−0.0086 (7)	−0.0068 (7)
C11	0.0319 (8)	0.0307 (8)	0.0314 (8)	−0.0008 (6)	−0.0067 (6)	−0.0019 (6)
C12	0.0208 (7)	0.0293 (7)	0.0295 (8)	−0.0024 (5)	−0.0024 (6)	−0.0018 (6)
C13	0.0279 (7)	0.0325 (8)	0.0326 (8)	−0.0001 (6)	−0.0055 (6)	−0.0003 (6)
C14	0.0310 (8)	0.0446 (9)	0.0284 (8)	0.0007 (7)	−0.0066 (6)	−0.0032 (7)
C15	0.0377 (9)	0.0415 (9)	0.0337 (9)	−0.0009 (7)	−0.0064 (7)	−0.0119 (7)
C16	0.0471 (10)	0.0299 (8)	0.0382 (9)	0.0008 (7)	−0.0089 (7)	−0.0065 (7)
P1	0.02439 (18)	0.02135 (18)	0.02522 (19)	−0.00115 (13)	−0.00411 (14)	−0.00261 (13)
C21	0.0320 (8)	0.0223 (7)	0.0270 (7)	−0.0013 (6)	−0.0051 (6)	−0.0019 (5)
C22	0.0360 (8)	0.0366 (8)	0.0334 (9)	0.0030 (7)	−0.0045 (7)	−0.0056 (7)
C23	0.0520 (11)	0.0497 (11)	0.0327 (9)	0.0048 (8)	0.0010 (8)	−0.0102 (8)
C24	0.0655 (13)	0.0507 (11)	0.0305 (9)	−0.0041 (9)	−0.0116 (9)	−0.0105 (8)
C25	0.0480 (10)	0.0427 (9)	0.0367 (9)	−0.0070 (8)	−0.0167 (8)	−0.0031 (7)
C26	0.0351 (8)	0.0298 (8)	0.0328 (8)	−0.0029 (6)	−0.0085 (7)	−0.0015 (6)
C31	0.0250 (7)	0.0228 (7)	0.0267 (7)	−0.0004 (5)	−0.0055 (6)	−0.0006 (5)
C32	0.0302 (7)	0.0243 (7)	0.0300 (8)	−0.0009 (6)	−0.0059 (6)	−0.0032 (6)
C33	0.0304 (8)	0.0265 (7)	0.0387 (9)	−0.0050 (6)	−0.0069 (7)	−0.0001 (6)
C34	0.0282 (7)	0.0319 (8)	0.0343 (8)	−0.0026 (6)	−0.0024 (6)	0.0038 (6)
C35	0.0327 (8)	0.0317 (8)	0.0269 (8)	0.0025 (6)	−0.0030 (6)	−0.0024 (6)
C36	0.0309 (8)	0.0251 (7)	0.0298 (8)	−0.0017 (6)	−0.0068 (6)	−0.0036 (6)
C41	0.0248 (7)	0.0259 (7)	0.0283 (7)	−0.0020 (6)	−0.0034 (6)	0.0006 (6)
C42	0.0341 (8)	0.0285 (8)	0.0381 (9)	0.0007 (6)	−0.0079 (7)	−0.0029 (6)
C43	0.0373 (9)	0.0323 (8)	0.0477 (10)	0.0078 (7)	−0.0054 (8)	0.0021 (7)
C44	0.0293 (8)	0.0461 (10)	0.0432 (10)	0.0018 (7)	−0.0080 (7)	0.0120 (8)
C45	0.0317 (8)	0.0474 (10)	0.0363 (9)	−0.0106 (7)	−0.0106 (7)	0.0058 (7)
C46	0.0322 (8)	0.0300 (8)	0.0322 (8)	−0.0062 (6)	−0.0055 (6)	−0.0001 (6)
C51	0.0300 (7)	0.0227 (7)	0.0250 (7)	−0.0019 (6)	−0.0059 (6)	−0.0019 (5)
C52	0.0297 (8)	0.0291 (8)	0.0331 (8)	−0.0018 (6)	−0.0042 (6)	−0.0003 (6)
C53	0.0395 (9)	0.0300 (8)	0.0365 (9)	−0.0104 (7)	−0.0078 (7)	0.0020 (6)
C54	0.0490 (10)	0.0229 (7)	0.0341 (8)	−0.0040 (7)	−0.0127 (7)	−0.0028 (6)
C55	0.0398 (9)	0.0273 (8)	0.0373 (9)	0.0045 (6)	−0.0097 (7)	−0.0065 (6)
C56	0.0299 (8)	0.0282 (7)	0.0324 (8)	−0.0013 (6)	−0.0059 (6)	−0.0039 (6)
O1A	0.0374 (6)	0.0347 (6)	0.0329 (6)	−0.0041 (5)	−0.0090 (5)	0.0023 (5)
B1A	0.0355 (9)	0.0288 (8)	0.0313 (9)	0.0030 (7)	−0.0116 (7)	−0.0043 (7)
F1A	0.0479 (6)	0.0391 (5)	0.0445 (6)	0.0096 (4)	−0.0151 (5)	0.0034 (4)
F2A	0.0525 (6)	0.0447 (6)	0.0326 (5)	−0.0058 (5)	−0.0103 (4)	−0.0106 (4)
C1A	0.0334 (8)	0.0255 (7)	0.0320 (8)	0.0011 (6)	−0.0080 (6)	−0.0071 (6)
C2A	0.0371 (8)	0.0233 (7)	0.0279 (8)	−0.0003 (6)	−0.0081 (6)	−0.0072 (6)
C3A	0.0514 (10)	0.0313 (8)	0.0287 (8)	−0.0047 (7)	−0.0117 (7)	−0.0048 (6)
C4A	0.0583 (11)	0.0386 (9)	0.0302 (9)	−0.0129 (8)	0.0026 (8)	−0.0069 (7)
C5A	0.0385 (9)	0.0481 (10)	0.0477 (11)	−0.0071 (8)	0.0033 (8)	−0.0126 (8)
C6A	0.0348 (9)	0.0384 (9)	0.0444 (10)	0.0019 (7)	−0.0082 (7)	−0.0065 (7)
C11A	0.0344 (8)	0.0278 (7)	0.0318 (8)	−0.0007 (6)	−0.0086 (6)	−0.0104 (6)
C12A	0.0359 (8)	0.0255 (7)	0.0304 (8)	0.0013 (6)	−0.0118 (6)	−0.0096 (6)
C13A	0.0447 (9)	0.0348 (8)	0.0383 (9)	0.0050 (7)	−0.0189 (8)	−0.0111 (7)
C14A	0.0422 (10)	0.0469 (10)	0.0543 (11)	0.0090 (8)	−0.0247 (9)	−0.0216 (9)
C15A	0.0319 (9)	0.0531 (11)	0.0588 (12)	−0.0012 (8)	−0.0126 (8)	−0.0290 (9)
C16A	0.0377 (9)	0.0399 (9)	0.0398 (9)	−0.0087 (7)	−0.0042 (7)	−0.0136 (7)
P1A	0.0303 (2)	0.02541 (19)	0.0278 (2)	0.00102 (15)	−0.00815 (15)	−0.00298 (15)
C21A	0.0310 (8)	0.0254 (7)	0.0314 (8)	−0.0003 (6)	−0.0079 (6)	−0.0017 (6)
C22A	0.0455 (10)	0.0404 (9)	0.0307 (9)	0.0091 (7)	−0.0093 (7)	−0.0040 (7)
C23A	0.0583 (12)	0.0509 (11)	0.0411 (10)	0.0184 (9)	−0.0198 (9)	−0.0010 (8)
C24A	0.0459 (10)	0.0419 (10)	0.0513 (11)	0.0160 (8)	−0.0116 (9)	−0.0055 (8)
C25A	0.0419 (9)	0.0347 (9)	0.0400 (9)	0.0045 (7)	−0.0032 (7)	−0.0075 (7)
C26A	0.0395 (9)	0.0297 (8)	0.0310 (8)	−0.0006 (6)	−0.0071 (7)	−0.0018 (6)
C31A	0.0386 (8)	0.0265 (7)	0.0353 (8)	−0.0017 (6)	−0.0162 (7)	−0.0022 (6)
C32A	0.0461 (10)	0.0307 (8)	0.0543 (11)	0.0032 (7)	−0.0227 (9)	−0.0073 (7)
C33A	0.0759 (14)	0.0244 (8)	0.0626 (13)	−0.0003 (8)	−0.0355 (11)	−0.0024 (8)
C34A	0.0721 (14)	0.0361 (10)	0.0525 (12)	−0.0190 (9)	−0.0242 (10)	0.0066 (8)
C35A	0.0535 (11)	0.0443 (10)	0.0489 (11)	−0.0154 (9)	−0.0101 (9)	0.0052 (8)
C36A	0.0427 (9)	0.0328 (8)	0.0413 (9)	−0.0048 (7)	−0.0089 (8)	0.0007 (7)
C41A	0.0321 (8)	0.0282 (7)	0.0287 (8)	−0.0029 (6)	−0.0072 (6)	−0.0013 (6)
C42A	0.0454 (9)	0.0273 (8)	0.0431 (10)	−0.0011 (7)	−0.0180 (8)	−0.0004 (7)
C43A	0.0519 (11)	0.0367 (9)	0.0557 (12)	0.0000 (8)	−0.0301 (9)	0.0020 (8)
C44A	0.0457 (10)	0.0421 (10)	0.0496 (11)	−0.0069 (8)	−0.0227 (8)	−0.0036 (8)
C45A	0.0459 (10)	0.0307 (8)	0.0524 (11)	−0.0079 (7)	−0.0170 (8)	−0.0031 (7)
C46A	0.0385 (9)	0.0272 (8)	0.0424 (9)	−0.0021 (6)	−0.0134 (7)	0.0003 (7)
C51A	0.0390 (9)	0.0409 (9)	0.0303 (8)	0.0125 (7)	−0.0087 (7)	−0.0054 (7)
C52A	0.0614 (13)	0.0625 (13)	0.0420 (11)	0.0127 (10)	−0.0167 (9)	−0.0194 (9)
C53A	0.093 (2)	0.0893 (18)	0.0426 (12)	0.0362 (16)	−0.0244 (13)	−0.0278 (12)
C54A	0.092 (2)	0.107 (2)	0.0323 (11)	0.0490 (17)	−0.0035 (12)	−0.0099 (13)
C55A	0.0646 (15)	0.108 (2)	0.0500 (14)	0.0099 (14)	0.0137 (12)	0.0074 (14)
C56A	0.0503 (11)	0.0681 (13)	0.0401 (11)	0.0011 (10)	0.0001 (9)	0.0005 (9)
N61	0.210 (4)	0.146 (3)	0.0611 (18)	−0.070 (3)	0.001 (2)	−0.0008 (18)
C62	0.147 (3)	0.099 (2)	0.0497 (16)	−0.032 (2)	−0.0075 (18)	−0.0033 (15)
C63	0.090 (3)	0.255 (6)	0.107 (3)	−0.002 (3)	0.003 (2)	0.050 (4)
N71	0.277 (5)	0.102 (2)	0.0662 (18)	0.009 (3)	−0.049 (2)	−0.0087 (17)
C72	0.125 (3)	0.101 (2)	0.0403 (13)	0.025 (2)	−0.0230 (15)	−0.0093 (14)
C73	0.096 (2)	0.109 (2)	0.079 (2)	0.0287 (18)	−0.0243 (17)	−0.0335 (18)
N81	0.101 (5)	0.151 (6)	0.160 (7)	−0.036 (4)	0.009 (4)	0.032 (5)
C82	0.061 (3)	0.083 (4)	0.143 (7)	−0.022 (3)	−0.010 (4)	0.034 (4)
N81'	0.081 (4)	0.140 (6)	0.139 (7)	−0.021 (4)	−0.039 (4)	0.003 (5)
C82'	0.050 (3)	0.087 (4)	0.103 (5)	−0.017 (3)	−0.019 (3)	−0.001 (4)
C83	0.114 (3)	0.096 (3)	0.154 (4)	−0.003 (2)	−0.041 (3)	0.013 (3)

### Bis(tetraphenylphosphonium) 6,6-difluoro-6H-dibenzo[c,e][1,2]oxaborinin-6-ide acetonitrile trisolvate (III) Geometric parameters (Å, º)

**Table:**

O1—C11	1.3492 (19)	C3A—H3A	0.9500
O1—B1	1.466 (2)	C4A—C5A	1.383 (3)
B1—F2	1.411 (2)	C4A—H4A	0.9500
B1—F1	1.412 (2)	C5A—C6A	1.380 (3)
B1—C1	1.603 (2)	C5A—H5A	0.9500
C1—C6	1.402 (2)	C6A—H6A	0.9500
C1—C2	1.407 (2)	C11A—C16A	1.403 (2)
C2—C3	1.404 (2)	C11A—C12A	1.411 (2)
C2—C12	1.480 (2)	C12A—C13A	1.405 (2)
C3—C4	1.383 (2)	C13A—C14A	1.377 (3)
C3—H3	0.9500	C13A—H13A	0.9500
C4—C5	1.385 (3)	C14A—C15A	1.384 (3)
C4—H4	0.9500	C14A—H14A	0.9500
C5—C6	1.385 (2)	C15A—C16A	1.382 (3)
C5—H5	0.9500	C15A—H15A	0.9500
C6—H6	0.9500	C16A—H16A	0.9500
C11—C16	1.397 (2)	P1A—C21A	1.7917 (15)
C11—C12	1.410 (2)	P1A—C31A	1.7930 (16)
C12—C13	1.401 (2)	P1A—C51A	1.7947 (17)
C13—C14	1.381 (2)	P1A—C41A	1.7966 (15)
C13—H13	0.9500	C21A—C22A	1.389 (2)
C14—C15	1.383 (2)	C21A—C26A	1.397 (2)
C14—H14	0.9500	C22A—C23A	1.384 (2)
C15—C16	1.378 (2)	C22A—H22A	0.9500
C15—H15	0.9500	C23A—C24A	1.381 (3)
C16—H16	0.9500	C23A—H23A	0.9500
P1—C51	1.7887 (14)	C24A—C25A	1.381 (3)
P1—C31	1.7935 (15)	C24A—H24A	0.9500
P1—C41	1.7944 (15)	C25A—C26A	1.379 (2)
P1—C21	1.7970 (15)	C25A—H25A	0.9500
C21—C22	1.394 (2)	C26A—H26A	0.9500
C21—C26	1.396 (2)	C31A—C36A	1.391 (2)
C22—C23	1.387 (2)	C31A—C32A	1.396 (2)
C22—H22	0.9500	C32A—C33A	1.392 (3)
C23—C24	1.380 (3)	C32A—H32A	0.9500
C23—H23	0.9500	C33A—C34A	1.376 (3)
C24—C25	1.381 (3)	C33A—H33A	0.9500
C24—H24	0.9500	C34A—C35A	1.371 (3)
C25—C26	1.385 (2)	C34A—H34A	0.9500
C25—H25	0.9500	C35A—C36A	1.384 (2)
C26—H26	0.9500	C35A—H35A	0.9500
C31—C36	1.400 (2)	C36A—H36A	0.9500
C31—C32	1.400 (2)	C41A—C42A	1.391 (2)
C32—C33	1.384 (2)	C41A—C46A	1.398 (2)
C32—H32	0.9500	C42A—C43A	1.384 (2)
C33—C34	1.384 (2)	C42A—H42A	0.9500
C33—H33	0.9500	C43A—C44A	1.386 (3)
C34—C35	1.388 (2)	C43A—H43A	0.9500
C34—H34	0.9500	C44A—C45A	1.381 (3)
C35—C36	1.385 (2)	C44A—H44A	0.9500
C35—H35	0.9500	C45A—C46A	1.385 (2)
C36—H36	0.9500	C45A—H45A	0.9500
C41—C46	1.393 (2)	C46A—H46A	0.9500
C41—C42	1.395 (2)	C51A—C56A	1.385 (3)
C42—C43	1.383 (2)	C51A—C52A	1.397 (3)
C42—H42	0.9500	C52A—C53A	1.384 (3)
C43—C44	1.381 (3)	C52A—H52A	0.9500
C43—H43	0.9500	C53A—C54A	1.371 (4)
C44—C45	1.382 (3)	C53A—H53A	0.9500
C44—H44	0.9500	C54A—C55A	1.373 (4)
C45—C46	1.384 (2)	C54A—H54A	0.9500
C45—H45	0.9500	C55A—C56A	1.397 (3)
C46—H46	0.9500	C55A—H55A	0.9500
C51—C52	1.396 (2)	C56A—H56A	0.9500
C51—C56	1.397 (2)	N61—C62	1.162 (5)
C52—C53	1.386 (2)	C62—C63	1.400 (5)
C52—H52	0.9500	C63—H63A	0.9800
C53—C54	1.386 (2)	C63—H63B	0.9800
C53—H53	0.9500	C63—H63C	0.9800
C54—C55	1.379 (2)	N71—C72	1.133 (4)
C54—H54	0.9500	C72—C73	1.401 (5)
C55—C56	1.387 (2)	C73—H73A	0.9800
C55—H55	0.9500	C73—H73B	0.9800
C56—H56	0.9500	C73—H73C	0.9800
O1A—C11A	1.3435 (19)	N81—C82	1.152 (11)
O1A—B1A	1.475 (2)	C82—C83	1.279 (9)
B1A—F2A	1.415 (2)	N81'—C82'	1.150 (10)
B1A—F1A	1.417 (2)	C82'—C83	1.488 (9)
B1A—C1A	1.601 (2)	C83—H83A	0.9800
C1A—C6A	1.398 (2)	C83—H83B	0.9800
C1A—C2A	1.408 (2)	C83—H83C	0.9800
C2A—C3A	1.408 (2)	C83—H83D	0.9800
C2A—C12A	1.479 (2)	C83—H83E	0.9800
C3A—C4A	1.381 (3)	C83—H83F	0.9800
			
C11—O1—B1	123.95 (13)	C4A—C3A—H3A	119.6
F2—B1—F1	105.79 (14)	C2A—C3A—H3A	119.6
F2—B1—O1	106.94 (14)	C3A—C4A—C5A	120.05 (17)
F1—B1—O1	107.56 (14)	C3A—C4A—H4A	120.0
F2—B1—C1	111.46 (14)	C5A—C4A—H4A	120.0
F1—B1—C1	112.30 (14)	C6A—C5A—C4A	119.53 (17)
O1—B1—C1	112.40 (13)	C6A—C5A—H5A	120.2
C6—C1—C2	117.90 (14)	C4A—C5A—H5A	120.2
C6—C1—B1	121.04 (14)	C5A—C6A—C1A	122.28 (17)
C2—C1—B1	121.05 (13)	C5A—C6A—H6A	118.9
C3—C2—C1	119.71 (14)	C1A—C6A—H6A	118.9
C3—C2—C12	121.62 (14)	O1A—C11A—C16A	117.06 (15)
C1—C2—C12	118.66 (13)	O1A—C11A—C12A	123.26 (14)
C4—C3—C2	120.81 (15)	C16A—C11A—C12A	119.67 (15)
C4—C3—H3	119.6	C13A—C12A—C11A	117.58 (15)
C2—C3—H3	119.6	C13A—C12A—C2A	122.52 (15)
C3—C4—C5	120.03 (15)	C11A—C12A—C2A	119.90 (14)
C3—C4—H4	120.0	C14A—C13A—C12A	122.25 (18)
C5—C4—H4	120.0	C14A—C13A—H13A	118.9
C6—C5—C4	119.52 (15)	C12A—C13A—H13A	118.9
C6—C5—H5	120.2	C13A—C14A—C15A	119.61 (17)
C4—C5—H5	120.2	C13A—C14A—H14A	120.2
C5—C6—C1	122.02 (16)	C15A—C14A—H14A	120.2
C5—C6—H6	119.0	C16A—C15A—C14A	120.04 (17)
C1—C6—H6	119.0	C16A—C15A—H15A	120.0
O1—C11—C16	116.71 (14)	C14A—C15A—H15A	120.0
O1—C11—C12	123.23 (14)	C15A—C16A—C11A	120.84 (18)
C16—C11—C12	120.06 (15)	C15A—C16A—H16A	119.6
C13—C12—C11	117.27 (14)	C11A—C16A—H16A	119.6
C13—C12—C2	122.68 (14)	C21A—P1A—C31A	108.71 (7)
C11—C12—C2	120.05 (14)	C21A—P1A—C51A	109.11 (7)
C14—C13—C12	122.13 (15)	C31A—P1A—C51A	108.93 (8)
C14—C13—H13	118.9	C21A—P1A—C41A	108.19 (7)
C12—C13—H13	118.9	C31A—P1A—C41A	112.38 (7)
C13—C14—C15	119.78 (15)	C51A—P1A—C41A	109.48 (8)
C13—C14—H14	120.1	C22A—C21A—C26A	120.21 (15)
C15—C14—H14	120.1	C22A—C21A—P1A	120.74 (12)
C16—C15—C14	119.73 (15)	C26A—C21A—P1A	119.00 (12)
C16—C15—H15	120.1	C23A—C22A—C21A	119.36 (16)
C14—C15—H15	120.1	C23A—C22A—H22A	120.3
C15—C16—C11	121.03 (16)	C21A—C22A—H22A	120.3
C15—C16—H16	119.5	C24A—C23A—C22A	120.20 (17)
C11—C16—H16	119.5	C24A—C23A—H23A	119.9
C51—P1—C31	111.54 (7)	C22A—C23A—H23A	119.9
C51—P1—C41	110.88 (7)	C25A—C24A—C23A	120.61 (16)
C31—P1—C41	107.22 (7)	C25A—C24A—H24A	119.7
C51—P1—C21	106.97 (7)	C23A—C24A—H24A	119.7
C31—P1—C21	110.05 (7)	C26A—C25A—C24A	119.84 (16)
C41—P1—C21	110.20 (7)	C26A—C25A—H25A	120.1
C22—C21—C26	120.23 (14)	C24A—C25A—H25A	120.1
C22—C21—P1	120.21 (12)	C25A—C26A—C21A	119.77 (15)
C26—C21—P1	119.04 (12)	C25A—C26A—H26A	120.1
C23—C22—C21	119.20 (16)	C21A—C26A—H26A	120.1
C23—C22—H22	120.4	C36A—C31A—C32A	119.58 (16)
C21—C22—H22	120.4	C36A—C31A—P1A	120.51 (12)
C24—C23—C22	120.38 (17)	C32A—C31A—P1A	119.90 (14)
C24—C23—H23	119.8	C33A—C32A—C31A	119.17 (19)
C22—C23—H23	119.8	C33A—C32A—H32A	120.4
C23—C24—C25	120.50 (17)	C31A—C32A—H32A	120.4
C23—C24—H24	119.8	C34A—C33A—C32A	120.51 (18)
C25—C24—H24	119.8	C34A—C33A—H33A	119.7
C24—C25—C26	120.00 (17)	C32A—C33A—H33A	119.7
C24—C25—H25	120.0	C35A—C34A—C33A	120.38 (18)
C26—C25—H25	120.0	C35A—C34A—H34A	119.8
C25—C26—C21	119.65 (16)	C33A—C34A—H34A	119.8
C25—C26—H26	120.2	C34A—C35A—C36A	120.1 (2)
C21—C26—H26	120.2	C34A—C35A—H35A	119.9
C36—C31—C32	119.94 (14)	C36A—C35A—H35A	119.9
C36—C31—P1	119.19 (11)	C35A—C36A—C31A	120.16 (17)
C32—C31—P1	120.49 (11)	C35A—C36A—H36A	119.9
C33—C32—C31	119.56 (14)	C31A—C36A—H36A	119.9
C33—C32—H32	120.2	C42A—C41A—C46A	120.00 (14)
C31—C32—H32	120.2	C42A—C41A—P1A	120.37 (12)
C32—C33—C34	120.22 (14)	C46A—C41A—P1A	119.52 (12)
C32—C33—H33	119.9	C43A—C42A—C41A	119.54 (15)
C34—C33—H33	119.9	C43A—C42A—H42A	120.2
C33—C34—C35	120.58 (15)	C41A—C42A—H42A	120.2
C33—C34—H34	119.7	C42A—C43A—C44A	120.58 (16)
C35—C34—H34	119.7	C42A—C43A—H43A	119.7
C36—C35—C34	119.87 (14)	C44A—C43A—H43A	119.7
C36—C35—H35	120.1	C45A—C44A—C43A	119.80 (16)
C34—C35—H35	120.1	C45A—C44A—H44A	120.1
C35—C36—C31	119.78 (14)	C43A—C44A—H44A	120.1
C35—C36—H36	120.1	C44A—C45A—C46A	120.52 (16)
C31—C36—H36	120.1	C44A—C45A—H45A	119.7
C46—C41—C42	120.02 (14)	C46A—C45A—H45A	119.7
C46—C41—P1	120.48 (12)	C45A—C46A—C41A	119.56 (15)
C42—C41—P1	118.99 (11)	C45A—C46A—H46A	120.2
C43—C42—C41	119.73 (15)	C41A—C46A—H46A	120.2
C43—C42—H42	120.1	C56A—C51A—C52A	120.02 (18)
C41—C42—H42	120.1	C56A—C51A—P1A	119.76 (14)
C44—C43—C42	120.06 (16)	C52A—C51A—P1A	120.03 (15)
C44—C43—H43	120.0	C53A—C52A—C51A	119.3 (2)
C42—C43—H43	120.0	C53A—C52A—H52A	120.3
C43—C44—C45	120.40 (15)	C51A—C52A—H52A	120.3
C43—C44—H44	119.8	C54A—C53A—C52A	120.8 (2)
C45—C44—H44	119.8	C54A—C53A—H53A	119.6
C44—C45—C46	120.28 (16)	C52A—C53A—H53A	119.6
C44—C45—H45	119.9	C53A—C54A—C55A	120.1 (2)
C46—C45—H45	119.9	C53A—C54A—H54A	120.0
C45—C46—C41	119.51 (15)	C55A—C54A—H54A	120.0
C45—C46—H46	120.2	C54A—C55A—C56A	120.5 (3)
C41—C46—H46	120.2	C54A—C55A—H55A	119.8
C52—C51—C56	120.30 (14)	C56A—C55A—H55A	119.8
C52—C51—P1	119.14 (11)	C51A—C56A—C55A	119.3 (2)
C56—C51—P1	119.98 (11)	C51A—C56A—H56A	120.3
C53—C52—C51	119.36 (15)	C55A—C56A—H56A	120.3
C53—C52—H52	120.3	N61—C62—C63	176.1 (5)
C51—C52—H52	120.3	C62—C63—H63A	109.5
C52—C53—C54	120.12 (15)	C62—C63—H63B	109.5
C52—C53—H53	119.9	H63A—C63—H63B	109.5
C54—C53—H53	119.9	C62—C63—H63C	109.5
C55—C54—C53	120.61 (14)	H63A—C63—H63C	109.5
C55—C54—H54	119.7	H63B—C63—H63C	109.5
C53—C54—H54	119.7	N71—C72—C73	178.1 (5)
C54—C55—C56	120.10 (15)	C72—C73—H73A	109.5
C54—C55—H55	119.9	C72—C73—H73B	109.5
C56—C55—H55	119.9	H73A—C73—H73B	109.5
C55—C56—C51	119.49 (14)	C72—C73—H73C	109.5
C55—C56—H56	120.3	H73A—C73—H73C	109.5
C51—C56—H56	120.3	H73B—C73—H73C	109.5
C11A—O1A—B1A	121.33 (13)	N81—C82—C83	177.7 (7)
F2A—B1A—F1A	106.56 (13)	N81'—C82'—C83	170.1 (8)
F2A—B1A—O1A	108.49 (14)	C82—C83—H83A	109.5
F1A—B1A—O1A	106.11 (13)	C82—C83—H83B	109.5
F2A—B1A—C1A	111.64 (13)	H83A—C83—H83B	109.5
F1A—B1A—C1A	112.18 (14)	C82—C83—H83C	109.5
O1A—B1A—C1A	111.55 (13)	H83A—C83—H83C	109.5
C6A—C1A—C2A	117.87 (15)	H83B—C83—H83C	109.5
C6A—C1A—B1A	121.82 (14)	C82'—C83—H83D	109.5
C2A—C1A—B1A	120.30 (14)	C82'—C83—H83E	109.5
C3A—C2A—C1A	119.49 (15)	H83D—C83—H83E	109.5
C3A—C2A—C12A	122.41 (14)	C82'—C83—H83F	109.5
C1A—C2A—C12A	118.10 (14)	H83D—C83—H83F	109.5
C4A—C3A—C2A	120.78 (16)	H83E—C83—H83F	109.5
			
C11—O1—B1—F2	132.26 (16)	C11A—O1A—B1A—F2A	−95.67 (16)
C11—O1—B1—F1	−114.49 (17)	C11A—O1A—B1A—F1A	150.16 (13)
C11—O1—B1—C1	9.6 (2)	C11A—O1A—B1A—C1A	27.71 (19)
F2—B1—C1—C6	52.6 (2)	F2A—B1A—C1A—C6A	−76.55 (19)
F1—B1—C1—C6	−65.9 (2)	F1A—B1A—C1A—C6A	43.0 (2)
O1—B1—C1—C6	172.65 (14)	O1A—B1A—C1A—C6A	161.88 (14)
F2—B1—C1—C2	−126.09 (15)	F2A—B1A—C1A—C2A	102.47 (16)
F1—B1—C1—C2	115.40 (16)	F1A—B1A—C1A—C2A	−138.00 (14)
O1—B1—C1—C2	−6.0 (2)	O1A—B1A—C1A—C2A	−19.10 (19)
C6—C1—C2—C3	−0.2 (2)	C6A—C1A—C2A—C3A	0.4 (2)
B1—C1—C2—C3	178.55 (14)	B1A—C1A—C2A—C3A	−178.69 (14)
C6—C1—C2—C12	−178.91 (13)	C6A—C1A—C2A—C12A	−179.02 (13)
B1—C1—C2—C12	−0.2 (2)	B1A—C1A—C2A—C12A	1.9 (2)
C1—C2—C3—C4	−0.1 (2)	C1A—C2A—C3A—C4A	0.0 (2)
C12—C2—C3—C4	178.53 (14)	C12A—C2A—C3A—C4A	179.37 (14)
C2—C3—C4—C5	0.3 (2)	C2A—C3A—C4A—C5A	−0.3 (2)
C3—C4—C5—C6	−0.1 (2)	C3A—C4A—C5A—C6A	0.2 (3)
C4—C5—C6—C1	−0.3 (3)	C4A—C5A—C6A—C1A	0.2 (3)
C2—C1—C6—C5	0.4 (2)	C2A—C1A—C6A—C5A	−0.5 (2)
B1—C1—C6—C5	−178.32 (15)	B1A—C1A—C6A—C5A	178.56 (16)
B1—O1—C11—C16	173.48 (16)	B1A—O1A—C11A—C16A	161.76 (14)
B1—O1—C11—C12	−6.8 (2)	B1A—O1A—C11A—C12A	−19.1 (2)
O1—C11—C12—C13	−179.84 (14)	O1A—C11A—C12A—C13A	179.16 (13)
C16—C11—C12—C13	−0.2 (2)	C16A—C11A—C12A—C13A	−1.7 (2)
O1—C11—C12—C2	−0.6 (2)	O1A—C11A—C12A—C2A	−1.2 (2)
C16—C11—C12—C2	179.06 (14)	C16A—C11A—C12A—C2A	177.91 (13)
C3—C2—C12—C13	4.3 (2)	C3A—C2A—C12A—C13A	9.5 (2)
C1—C2—C12—C13	−176.98 (14)	C1A—C2A—C12A—C13A	−171.16 (14)
C3—C2—C12—C11	−174.85 (14)	C3A—C2A—C12A—C11A	−170.15 (14)
C1—C2—C12—C11	3.8 (2)	C1A—C2A—C12A—C11A	9.2 (2)
C11—C12—C13—C14	0.7 (2)	C11A—C12A—C13A—C14A	0.8 (2)
C2—C12—C13—C14	−178.52 (14)	C2A—C12A—C13A—C14A	−178.82 (14)
C12—C13—C14—C15	−0.8 (2)	C12A—C13A—C14A—C15A	0.5 (2)
C13—C14—C15—C16	0.4 (3)	C13A—C14A—C15A—C16A	−0.8 (3)
C14—C15—C16—C11	0.1 (3)	C14A—C15A—C16A—C11A	−0.2 (2)
O1—C11—C16—C15	179.50 (16)	O1A—C11A—C16A—C15A	−179.37 (14)
C12—C11—C16—C15	−0.2 (3)	C12A—C11A—C16A—C15A	1.5 (2)
C51—P1—C21—C22	88.88 (14)	C31A—P1A—C21A—C22A	−107.92 (14)
C31—P1—C21—C22	−149.78 (12)	C51A—P1A—C21A—C22A	10.75 (16)
C41—P1—C21—C22	−31.74 (14)	C41A—P1A—C21A—C22A	129.78 (14)
C51—P1—C21—C26	−82.85 (13)	C31A—P1A—C21A—C26A	69.37 (14)
C31—P1—C21—C26	38.49 (14)	C51A—P1A—C21A—C26A	−171.96 (13)
C41—P1—C21—C26	156.53 (12)	C41A—P1A—C21A—C26A	−52.93 (14)
C26—C21—C22—C23	−2.1 (2)	C26A—C21A—C22A—C23A	0.3 (3)
P1—C21—C22—C23	−173.71 (13)	P1A—C21A—C22A—C23A	177.55 (15)
C21—C22—C23—C24	0.9 (3)	C21A—C22A—C23A—C24A	−1.1 (3)
C22—C23—C24—C25	0.8 (3)	C22A—C23A—C24A—C25A	0.7 (3)
C23—C24—C25—C26	−1.4 (3)	C23A—C24A—C25A—C26A	0.6 (3)
C24—C25—C26—C21	0.2 (3)	C24A—C25A—C26A—C21A	−1.4 (3)
C22—C21—C26—C25	1.5 (2)	C22A—C21A—C26A—C25A	1.0 (2)
P1—C21—C26—C25	173.25 (12)	P1A—C21A—C26A—C25A	−176.32 (13)
C51—P1—C31—C36	−38.09 (14)	C21A—P1A—C31A—C36A	−14.99 (16)
C41—P1—C31—C36	83.48 (13)	C51A—P1A—C31A—C36A	−133.78 (14)
C21—P1—C31—C36	−156.66 (11)	C41A—P1A—C31A—C36A	104.73 (14)
C51—P1—C31—C32	149.03 (12)	C21A—P1A—C31A—C32A	164.34 (13)
C41—P1—C31—C32	−89.40 (13)	C51A—P1A—C31A—C32A	45.56 (15)
C21—P1—C31—C32	30.46 (14)	C41A—P1A—C31A—C32A	−75.94 (15)
C36—C31—C32—C33	2.1 (2)	C36A—C31A—C32A—C33A	0.8 (3)
P1—C31—C32—C33	174.95 (12)	P1A—C31A—C32A—C33A	−178.57 (14)
C31—C32—C33—C34	−0.1 (2)	C31A—C32A—C33A—C34A	1.2 (3)
C32—C33—C34—C35	−1.5 (2)	C32A—C33A—C34A—C35A	−2.0 (3)
C33—C34—C35—C36	1.2 (2)	C33A—C34A—C35A—C36A	0.8 (3)
C34—C35—C36—C31	0.8 (2)	C34A—C35A—C36A—C31A	1.2 (3)
C32—C31—C36—C35	−2.4 (2)	C32A—C31A—C36A—C35A	−1.9 (3)
P1—C31—C36—C35	−175.36 (11)	P1A—C31A—C36A—C35A	177.39 (14)
C51—P1—C41—C46	29.64 (15)	C21A—P1A—C41A—C42A	157.16 (14)
C31—P1—C41—C46	−92.35 (13)	C31A—P1A—C41A—C42A	37.13 (16)
C21—P1—C41—C46	147.88 (12)	C51A—P1A—C41A—C42A	−84.04 (15)
C51—P1—C41—C42	−158.56 (12)	C21A—P1A—C41A—C46A	−26.78 (16)
C31—P1—C41—C42	79.45 (14)	C31A—P1A—C41A—C46A	−146.80 (13)
C21—P1—C41—C42	−40.32 (14)	C51A—P1A—C41A—C46A	92.02 (15)
C46—C41—C42—C43	−1.5 (2)	C46A—C41A—C42A—C43A	−0.1 (3)
P1—C41—C42—C43	−173.39 (13)	P1A—C41A—C42A—C43A	175.99 (15)
C41—C42—C43—C44	1.1 (3)	C41A—C42A—C43A—C44A	0.1 (3)
C42—C43—C44—C45	0.1 (3)	C42A—C43A—C44A—C45A	0.3 (3)
C43—C44—C45—C46	−0.8 (3)	C43A—C44A—C45A—C46A	−0.7 (3)
C44—C45—C46—C41	0.3 (2)	C44A—C45A—C46A—C41A	0.7 (3)
C42—C41—C46—C45	0.9 (2)	C42A—C41A—C46A—C45A	−0.4 (3)
P1—C41—C46—C45	172.58 (12)	P1A—C41A—C46A—C45A	−176.43 (14)
C31—P1—C51—C52	160.72 (12)	C21A—P1A—C51A—C56A	91.18 (16)
C41—P1—C51—C52	41.29 (14)	C31A—P1A—C51A—C56A	−150.29 (15)
C21—P1—C51—C52	−78.89 (13)	C41A—P1A—C51A—C56A	−27.04 (17)
C31—P1—C51—C56	−28.00 (14)	C21A—P1A—C51A—C52A	−83.80 (16)
C41—P1—C51—C56	−147.42 (12)	C31A—P1A—C51A—C52A	34.73 (17)
C21—P1—C51—C56	92.39 (13)	C41A—P1A—C51A—C52A	157.98 (14)
C56—C51—C52—C53	1.3 (2)	C56A—C51A—C52A—C53A	−0.3 (3)
P1—C51—C52—C53	172.52 (12)	P1A—C51A—C52A—C53A	174.66 (16)
C51—C52—C53—C54	−0.5 (2)	C51A—C52A—C53A—C54A	0.3 (3)
C52—C53—C54—C55	−0.3 (2)	C52A—C53A—C54A—C55A	0.2 (4)
C53—C54—C55—C56	0.2 (2)	C53A—C54A—C55A—C56A	−0.5 (4)
C54—C55—C56—C51	0.6 (2)	C52A—C51A—C56A—C55A	−0.1 (3)
C52—C51—C56—C55	−1.3 (2)	P1A—C51A—C56A—C55A	−175.04 (17)
P1—C51—C56—C55	−172.51 (12)	C54A—C55A—C56A—C51A	0.5 (4)

### Bis(tetraphenylphosphonium) 6,6-difluoro-6H-dibenzo[c,e][1,2]oxaborinin-6-ide acetonitrile trisolvate (III) Hydrogen-bond geometry (Å, º)

**Table:**

*D*—H···*A*	*D*—H	H···*A*	*D*···*A*	*D*—H···*A*
C26*A*—H26*A*···O1*A*	0.95	2.58	3.322 (2)	135
C32*A*—H32*A*···F2^i^	0.95	2.40	3.069 (2)	127
C42*A*—H42*A*···F2^i^	0.95	2.51	3.378 (2)	152
C44*A*—H44*A*···F1*A*^i^	0.95	2.54	3.482 (2)	172
C53—H53···O1^i^	0.95	2.59	3.389 (2)	142
C54*A*—H54*A*···F2^ii^	0.95	2.55	3.436 (3)	156
C55*A*—H55*A*···N81′^i^	0.95	2.52	3.180 (8)	127
C63—H63*A*···N61^iii^	0.98	2.58	3.365 (6)	137
C63—H63*C*···N71^iv^	0.98	2.47	3.371 (7)	153
C73—H73*A*···F1	0.98	2.19	3.106 (4)	156
C73—H73*B*···N81^ii^	0.98	2.45	3.313 (10)	146
C83—H83*A*···F1	0.98	2.52	3.252 (4)	131

Symmetry codes: (i) *x*+1, *y*, *z*; (ii) −*x*+1, −*y*+1, −*z*; (iii) −*x*+2, −*y*+2, −*z*; (iv) *x*+1, *y*+1, *z*.
